# Nonsense-mediated mRNA decay uses complementary mechanisms to suppress mRNA and protein accumulation

**DOI:** 10.26508/lsa.202101217

**Published:** 2021-12-08

**Authors:** Dylan B Udy, Robert K Bradley

**Affiliations:** 1 Computational Biology Program, Public Health Sciences Division, Fred Hutchinson Cancer Research Center, Seattle, WA, USA; 2 Basic Sciences Division, Fred Hutchinson Cancer Research Center, Seattle, WA, USA; 3 Molecular and Cellular Biology Graduate Program, University of Washington, Seattle, WA, USA

## Abstract

Nonsense-mediated mRNA decay (NMD) is an essential, highly conserved quality control pathway that detects and degrades mRNAs containing premature termination codons. Although the essentiality of NMD is frequently ascribed to its prevention of truncated protein accumulation, the extent to which NMD actually suppresses proteins encoded by NMD-sensitive transcripts is less well-understood than NMD-mediated suppression of mRNA. Here, we describe a reporter system that permits accurate quantification of both mRNA and protein levels via stable integration of paired reporters encoding NMD-sensitive and NMD-insensitive transcripts into the AAVS1 safe harbor loci in human cells. We use this system to demonstrate that NMD suppresses proteins encoded by NMD-sensitive transcripts by up to eightfold more than the mRNA itself. Our data indicate that NMD limits the accumulation of proteins encoded by NMD substrates by mechanisms beyond mRNA degradation, such that even when NMD-sensitive mRNAs escape destruction, their encoded proteins are still effectively suppressed.

## Abstract (precis)

A reporter system for quantitative measurements of NMD-sensitive mRNA and protein levels in mammalian cells shows that NMD suppresses protein levels to a greater degree than RNA levels.

## Introduction

Nonsense-mediated mRNA decay (NMD) is a eukaryotic cellular surveillance system that acts to prevent the accumulation of potentially deleterious truncated proteins by targeting mRNAs with premature termination codons (PTCs) for degradation (seminal articles: Chang et al [1979], Losson and Lacroute [1979], Maquat et al [1981], and Kinniburgh et al [1982]; reviewed in: Lykke-Andersen and Jensen [2015] and Kurosaki et al [2019]). In mammalian cells, mRNAs with a PTC upstream of an exon–exon junction are recognized as aberrant during translation through the interaction of the terminating ribosome with an exon junction complex (EJC) that is deposited upstream of a splice junction (Nagy & Maquat, 1998; Le Hir et al, 2000, 2001, 2016; Lykke-Andersen et al, 2001; Schlautmann & Gehring, 2020). This leads to recruitment of RNA degradation machinery that cleaves the mRNA (Huntzinger et al, 2008; Eberle et al, 2009) and thus prevents continued production of truncated proteins.

Truncated proteins derived from NMD-insensitive transcripts, in which the PTC resides in the last exon or last ∼55 nucleotides of the penultimate exon, can cause disease in heterozygotes, whereas heterozygous individuals bearing PTCs that generate NMD-sensitive transcripts in the same genes are often unaffected (Holbrook et al, 2004; Khajavi et al, 2006; Miller & Pearce, 2014; Coban-Akdemir et al, 2018). These genetic findings strongly support the hypothesis that limiting potentially deleterious truncated protein accumulation is essential for cell health and homeostasis and likely one of the primary selection pressures for evolution and maintenance of the NMD pathway. Despite this hypothesized importance, levels of proteins encoded by NMD-sensitive transcripts have not been quantitatively measured to the same extent as corresponding mRNA levels. Levels of NMD-sensitive mRNAs have been extensively measured and characterized (Zhang et al, 1998; Mendell et al, 2004; Tani et al, 2012; Lindeboom et al, 2016; Celik et al, 2017; Colombo et al, 2017; Kurosaki et al, 2018; Karousis et al, 2021; Kovalak et al, 2021), clearly demonstrating that NMD suppresses mRNA levels. These reduced mRNA levels imply coincident reduction of corresponding protein levels. However, in the absence of highly quantitative protein-level measurements, the extent to which protein versus mRNA alone is suppressed remains unclear.

There are multiple lines of evidence supporting the idea that proteins encoded by NMD-sensitive transcripts have the potential to accumulate to non-negligible levels: (1) NMD is a translation dependent process, so production of some potentially deleterious proteins is required to degrade the mRNA; (2) NMD does not completely deplete NMD-sensitive transcripts from cells—some remain at 20–35% levels of corresponding NMD-insensitive transcripts (Trcek et al, 2013; Hoek et al, 2019); (3) there is evidence for a subpopulation of NMD-sensitive mRNAs that are as stable as NMD-insensitive mRNAs (Tani et al, 2012; Trcek et al, 2013; Hoek et al, 2019); (4) NMD transcripts can be translated multiple times and degradation can occur after the pioneer round of translation (Kurosaki et al, 2018; Hoek et al, 2019); (5) NMD transcripts have been found to be associated with polysomes (Kim et al, 2017; Kurosaki et al, 2018); (6) NMD transcripts can be targeted for degradation even after associating with the eIF4F complex that is involved in bulk protein synthesis (Durand & Lykke-Andersen, 2013; Rufener & Mühlemann, 2013); and (7) select proteins encoded by endogenous transcripts that are predicted to be targeted by NMD can be detected (Giorgi et al, 2007).

Much of the previous work on NMD has used reporter systems (Daar & Maquat, 1988; Carter et al, 1996; Zhang et al, 1998; Bühler et al, 2004; Eberle et al, 2008; Kuroha et al, 2009; Kim et al, 2017; Hoek et al, 2019) that facilitate changes to the reporter sequence to test various features (e.g., PTC location, NMD-inducing features, and 3′-UTR length) in a controlled manner and precisely quantify how such features affect mRNA levels. Some systems use protein-level measurements from NMD-sensitive reporters using fluorescent proteins or luciferase (Paillusson et al, 2005; Boelz et al, 2006; Nickless et al, 2014; Pereverzev et al, 2015; Alexandrov et al, 2017; Baird et al, 2018; Sato & Singer, 2021 *Preprint*; Cheruiyot et al, 2021; D’Orazio et al, 2021 *Preprint*; Chu et al, 2021; Zinshteyn et al, 2021), but these past studies have not directly compared mRNA and protein levels. Several studies have measured both mRNA and protein levels from NMD-sensitive reporters in yeast (Muhlrad & Parker, 1999; Kuroha et al, 2009) and human cells (Inoue et al, 2004; Boelz et al, 2006; Anczuków et al, 2008; Kang et al, 2009; Kim et al, 2017; Aksit et al, 2019), although the reporters used in human cells were not optimized for precise protein-level measurements. Intriguingly, studies in yeast indicated that protein levels can be reduced to a greater degree than mRNA levels (Muhlrad & Parker, 1999; Kuroha et al, 2009). However, in the absence of quantitative -and simultaneous measurements of levels of NMD-sensitive mRNAs and their encoded proteins in human cells, the extent to which mRNA and protein suppression contribute to the overall suppression of gene expression by NMD remains unclear. Overall, these past studies highlight the need to develop NMD reporters with quantitative readouts that are suitable for use in mammalian cells.

We therefore sought to develop a system to make quantitative mRNA- and protein-level measurements in human cells to systematically determine how NMD sensitivity influences levels of the encoded proteins relative to their parent mRNAs.

## Results

### Development of an NMD reporter system for precise quantification of mRNA and protein levels

We sought to develop a reporter system based on previously validated reporters that included new, complementary features which facilitated precise measurement of both mRNA and protein levels. Such features include (1) protein-level measurement with a high dynamic range; (2) full-length protein domains to minimize inherent instability of a truncated protein lacking any folded domains as a potentially confounding source of variability between proteins encoded by NMD-insensitive (“control”) and NMD-sensitive (“NMD(+)”) reporters; (3) internally included, NMD-insensitive control reporters to permit accurate normalization between samples; (4) straightforward measurement of mRNA and protein stability; and (5) stable integration into a “safe harbor” genomic locus to eliminate the need for repeated transient transfections—which itself can reduce NMD efficiency (Gerbracht et al, 2017)—as well as remove stochastic location of genomic integration as a potentially confounding source of variability between experiments.

**Figure 1.**
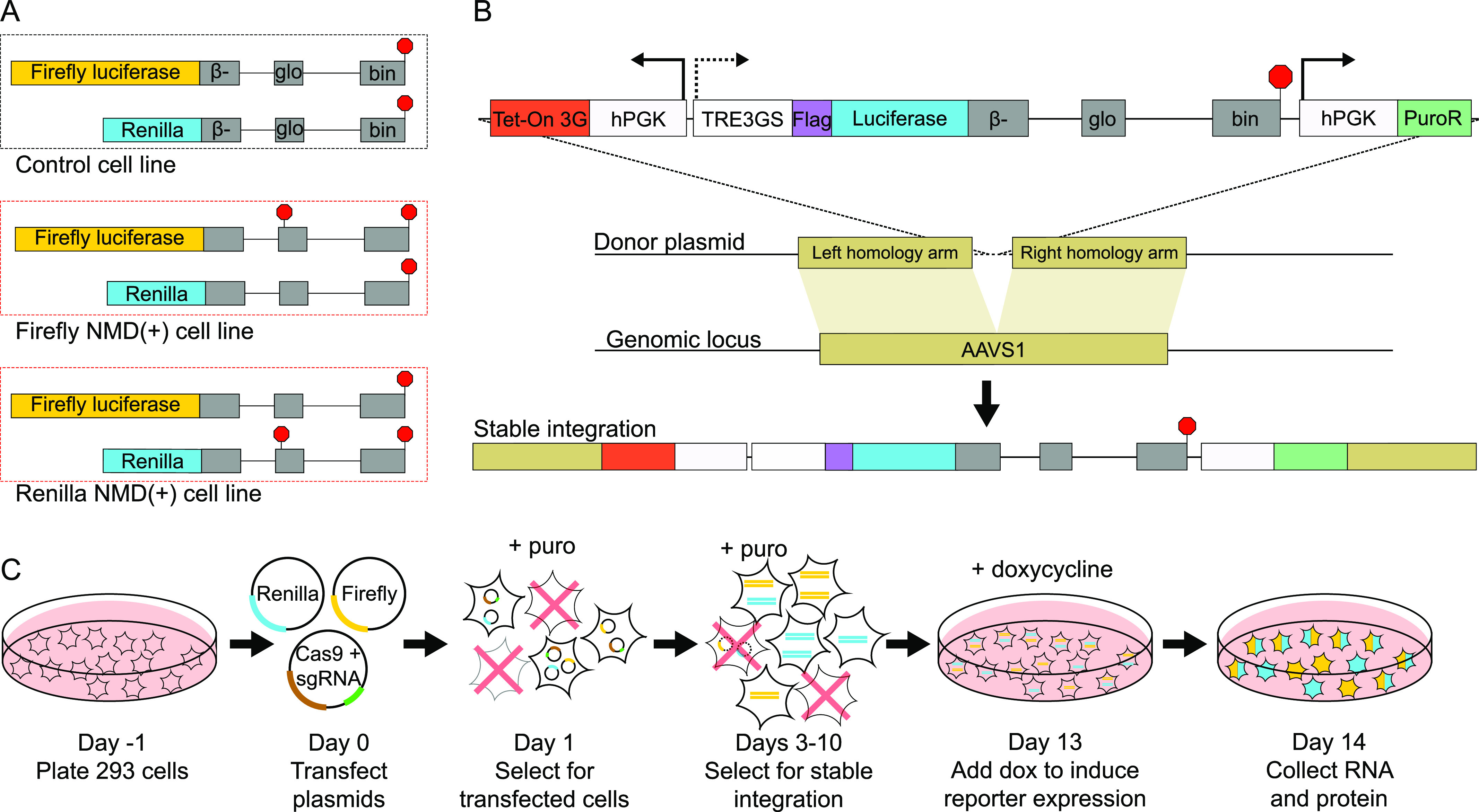
Development of a reporter system for quantitative mRNA- and protein-level measurements. **(A)** Diagrams of the luciferase-based NMD reporters used in this study. The reporters were grouped in pairs (one firefly luciferase reporter and one Renilla luciferase reporter) and used together in a control cell line (both reporters have a normal termination codon) or NMD(+) cell lines (one reporter with normal TC and the other with a premature termination codon). **(B)** Schematic of the reporter plasmid sequence that was stably integrated into the AAVS1 loci of 293 cells. **(C)** Workflow describing how the NMD reporters were stably integrated into 293 cells using CRISPR-Cas9 genome engineering and how selection for only cells with stably integrated reporters was performed.

We employed luciferase-based reporters (based on previously published and validated reporters; Baird et al, 2018) to achieve high dynamic range protein-level measurements from reporter proteins with a full-length, functional domain (Fig 1A). Using luminescence as the readout precludes the need for Western blotting and antibodies, eliminating additional potentially confounding variables. The luciferase sequences are followed by sequences that code for either full-length β-globin (control reporters) or truncated β-globin with a PTC at amino acid position 39 (NMD(+) reporters) (Fig 1A) (Zhang et al, 1998; Baird et al, 2018).

**Figure S1.**
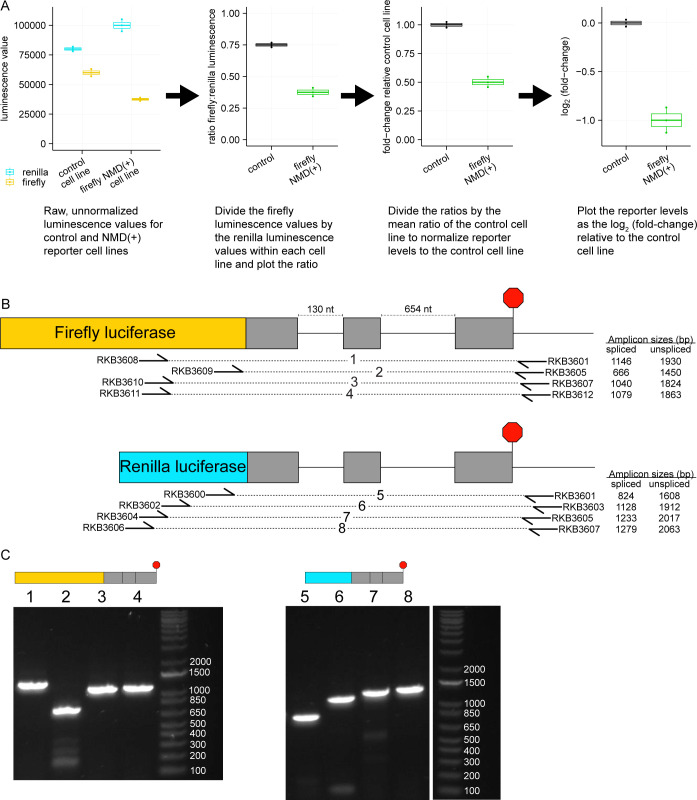
Stably integrated luciferase reporters are spliced correctly. **(A)** Mock dual-luciferase assay data demonstrating how to normalize the luminescence values of the luciferases and how to plot the level of the NMD(+) reporter relative to the control reporter. **(B)** Schematics showing how primers were designed for each luciferase reporter to confirm correct splicing. The forward primers were designed in the luciferase sequence, whereas the reverse primers were designed in the 3′-UTR; if either intron were not spliced out there would be a noticeable size shift in the PCR amplicon. The sizes are shown for the expected PCR amplicons for each primer set from either the fully spliced reporter mRNA or the fully unspliced reporter mRNA. The primer sequences are listed in Table S1 – Key resources table. **(B, C)** End point RT-PCR using the primer sets shown in (B) and cDNA from cell lines with stably integrated luciferase reporters. The number above each lane corresponds to the primer pair used for that PCR (listed in (B)). Source data are available for this figure.

We took advantage of the reporters’ potentiality for use in a dual-luciferase system (Sherf et al, 1996) in which two distinct luciferase enzymes (firefly and Renilla) are co-expressed and one is designated as an internal control (Fig 1A), permitting normalization between samples with the same internal control luciferase. For example, the firefly NMD(+) reporter is normalized to a Renilla control reporter in the same sample, and that ratio is then compared with the firefly control reporter normalized to the Renilla control reporter in another sample to determine the firefly NMD(+) reporter level relative to the firefly control reporter level (Fig S1A). We created two distinct NMD(+) cell lines, in which either firefly or Renilla luciferase is used in the NMD(+) reporter, whereas the other luciferase is used in the control reporter, and vice versa (Fig 1A, bottom two sets of reporters). This strategy ensured that results were dependent on the NMD sensitivity of the reporter rather than specific to a particular luciferase.

RNA stability is often measured using actinomycin D to inhibit transcription, which can lead to widespread changes in the transcriptome and pleiotropic effects on cell function (Lugowski et al, 2018). We therefore used a Tet-On inducible promoter system (Gossen et al, 1995; Heinz et al, 2011) with our NMD reporters (Fig 1B) to modulate reporter expression with doxycycline, enabling temporal control of expression and mRNA stability measurements without bulk transcription inhibition.

Finally, mRNAs transcribed from transiently transfected reporters are not efficiently degraded by NMD in some cell types (Gerbracht et al, 2017). To avoid such a disruptive complication and obtain more uniform and consistent reporter expression, we used CRISPR/Cas9-mediated genome engineering to stably integrate the reporters into the AAVS1 safe harbor loci in HEK-293 cells. The reporter sequences were cloned into a donor plasmid with homology arms to the AAVS1 locus (Natsume et al, 2016) (Fig 1B), and the donor plasmids were co-transfected with a Cas9/AAVS1-sgRNA expressing plasmid into HEK-293 cells. Cells with stably integrated reporters were selected for using puromycin over several days (workflow in Fig 1C). After generation of these stable cell lines, we used RT-PCR to confirm that these reporters were efficiently and correctly spliced (Fig S1B and C). We performed all subsequent experiments with these cell lines unless described otherwise.

### mRNA levels and decay kinetics confirm NMD sensitivity of the reporters

**Figure 2.**
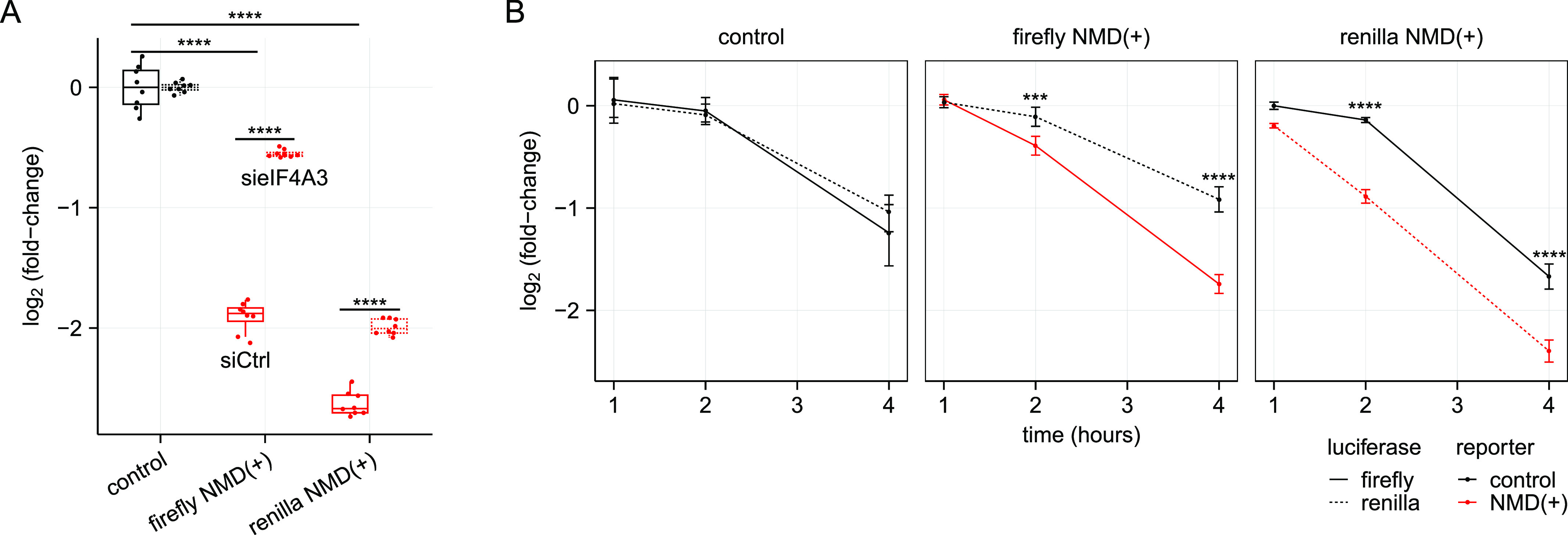
NMD reporter steady-state mRNA levels and decay kinetics are comparable with those of previously published NMD-sensitive reporters. **(A)** Box plots showing the steady-state NMD reporter mRNA levels relative to the levels in the control cell lines with and without NMD inhibition via RNAi-mediated eIF4A3 depletion. Each box plot shows n = 4 technical replicates from n = 2 biological replicates for a total of eight data points. An unpaired two-samples *t* test was used for calculating the *P*-values (****P < 0.0001, exact values listed in Table S2). Median values for each of the box plots are provided in Table S3 as percentages. **(B)** Line plots showing the decay kinetics of the NMD reporter mRNA after doxycycline removal to turn off reporter transcription. The firefly and Renilla reporters are plotted as separate lines. The levels at each time point are plotted relative to the levels at time point 0. The “control” panel is a combination of two independent control cell lines (both cell lines have the same two control reporters integrated, but the lines were generated separately with CRISPR/Cas9 mediated genome engineering). Each time point corresponds to n = 4 technical replicates (n = 2 biological replicates for the control panel, n = 8 data points), with error bars showing the range of those values and the line plot connecting at the mean of the values. An unpaired two-samples t-test was used for calculating the *P*-values (***P < 0.001, ****P < 0.0001, exact values listed in Table S2), which used the ratio of individual firefly replicate values to the mean Renilla value at each time point for the NMD(+) reporter cell lines compared to the ratios at the same time point in the control cell lines. The data is plotted starting at 1 h after doxycycline removal because there is little change in the reporter levels between the 0- and 1-h time points, likely due to technical limitations of the dox-inducible promoter.

We first validated our reporters by confirming that they were subject to RNA degradation by the NMD machinery. We measured reporter mRNA levels via qRT-PCR and found that the NMD(+) reporter mRNA levels were reduced to ∼15–25% of the corresponding control reporter mRNA levels (Fig 2A, “siCtrl” solid boxes), a reduction similar to that observed in previous experiments that used β-globin reporters with a PTC at amino acid position 39 (Zhang et al, 1998). We used poly-dT primers for cDNA synthesis (additional details in the Materials and Methods section) to select for mature polyadenylated mRNA and decrease the likelihood that our samples contain substantial fractions of nascent or not fully processed mRNA; however, we cannot rule out the possibility that some amount of nuclear RNA is measured in this assay.

**Figure S2.**
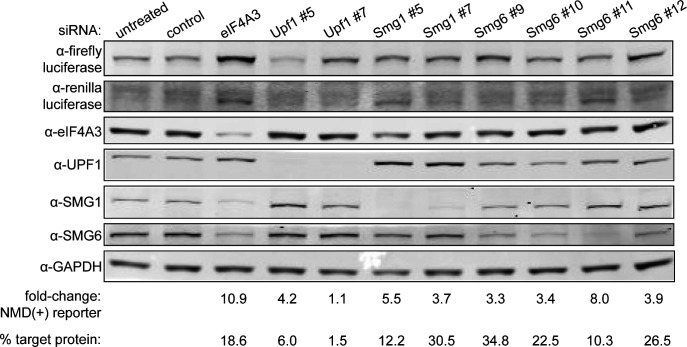
NMD factors are depleted upon RNAi treatment. Western blot showing the depletion of several factors involved in NMD and the corresponding change in Renilla NMD(+) reporter protein levels in samples from the polyclonal Renilla NMD(+) cell line. The fold-change at the bottom represents the change in intensity of the Renilla NMD(+) reporter protein band in each lane relative to the intensity of the band in the untreated control lane. The “% target protein” refers to the level of depleted protein remaining in each lane for the specific factor that was targeted by RNAi in that sample. The number after the gene name at the top of each lane corresponds to the specific siRNA used; additional siRNA details are listed in Table S1 – Key resources table. The order of the siRNAs for each gene in the Western blot corresponds to the order of the box plots for each gene shown in Fig 3A. Source data are available for this figure.

To confirm that the lower mRNA levels of the NMD(+) reporters are a consequence of the desired NMD sensitivity of the transcript, we inhibited NMD by depleting eIF4A3 (Fig S2). eIF4A3 is a core component of the EJC (Chan et al, 2004; Palacios et al, 2004; Shibuya et al, 2004; Ferraiuolo et al, 2004) and binds directly to both spliced RNA and other core EJC factors (Shibuya et al, 2004; Bono et al, 2006; Andersen et al, 2006). Depletion of eIF4A3 is predicted to reduce EJC deposition on spliced RNAs and leads to preferential stabilization of NMD-sensitive transcripts (Palacios et al, 2004; Shibuya et al, 2004; Ferraiuolo et al, 2004; Giorgi et al, 2007). NMD(+) reporter mRNA levels increased with eIF4A3 depletion (Fig 2A, “sieIF4A3” dashed boxes) by up to ∼3-fold relative to control siRNA samples, confirming that reduced steady-state levels arise from action of the NMD machinery. Although eIF4A3 depletion could potentially have unintended effects on other aspects of cell physiology given the multifunctionality of the EJC (Le Hir et al, 2016; Ye et al, 2021), our use of both control (NMD-insensitive) and NMD(+) reporter cell lines in the knockdown experiments and subsequent normalization of NMD(+) reporter mRNA levels to control reporters and the control cell line make these data robust to such effects.

To determine if faster RNA degradation was responsible for the observed lower steady-state NMD(+) reporter mRNA levels, we turned off transcription using the inducible promoter to directly measure reporter mRNA decay kinetics. The NMD(+) reporter mRNA was degraded faster than was the control reporter mRNA (Fig 2B), as expected and consistent with previous studies (Trcek et al, 2013; Kim et al, 2017; Aksit et al, 2019). We observed faster degradation for both the firefly and Renilla NMD(+) reporters (Fig 2B, right two panels), although there were modest differences in the magnitudes of the changes. We observed these differences in magnitude for both the steady-state mRNA levels and mRNA degradation rates (Fig 2A and B), suggesting that they may arise from the different luciferase CDSs used in each reporter. These CDS-specific differences highlight the importance of controlling for CDS identity when studying NMD, a control that is inherent to our reporter system given its use of CDS-matched NMD-sensitive and NMD-insensitive transcripts.

Overall, these data confirm that our stably integrated reporters are modulated by NMD at the RNA level and that NMD activity suppresses their steady-state levels and influences their decay kinetics as expected based on results from previously published NMD reporters.

### NMD(+) reporter protein levels are reduced to a greater degree than are mRNA levels

We next took advantage of the reporters’ luminescence to make precise and quantitative measurements of protein levels. We inhibited NMD with RNAi of multiple NMD factors and qualitatively assessed changes in Renilla NMD(+) reporter protein levels via Western blot (Fig S2). We observed effective protein depletion with at least one siRNA for each NMD factor. In control samples, we observed a very faint band corresponding to the Renilla luciferase plus truncated β-globin fusion protein (Fig S2, lanes 1–2). The band intensity increased to the greatest degree with depletion of eIF4A3 (Fig S2, lane 3). In general, the greater degree of protein depletion for each NMD factor corresponded with a greater degree of increase in signal intensity from the Renilla NMD(+) reporter protein band (Fig S2, lanes 6 and 10).

**Figure 3.**
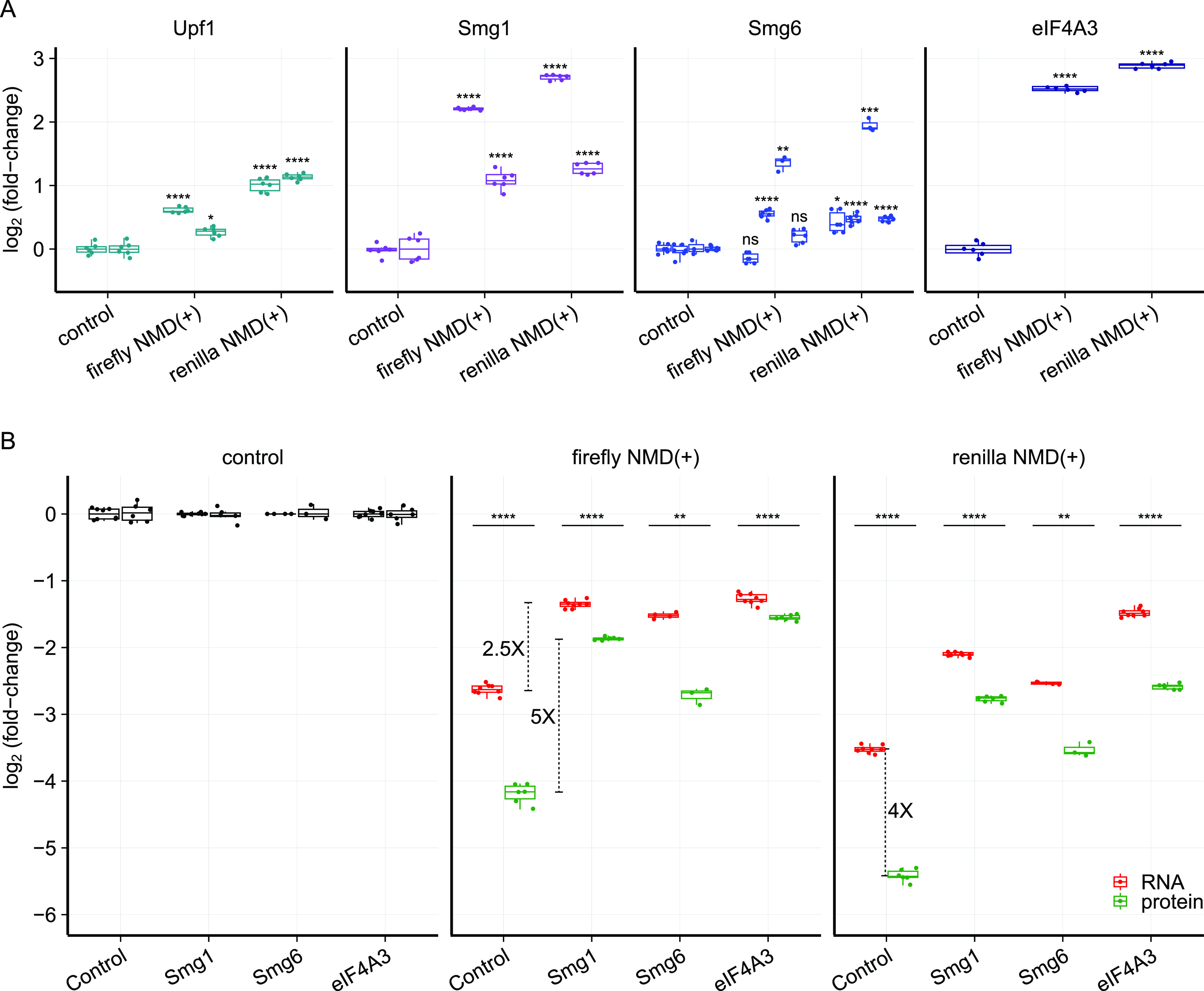
NMD(+) reporter protein levels are reduced relative to control reporter protein levels and to a greater degree than NMD(+) reporter mRNA levels. **(A)** Box plots showing the increase in NMD(+) reporter protein levels relative to control reporter protein levels upon depletion of NMD factors UPF1, SMG1, SMG6, and eIF4A3. The dual-luciferase assay was used to measure reporter protein levels. Each box plot shows n = 3 technical replicates normalized to n = 2 biological replicates for a total of six data points. The specific siRNAs used are listed in Table S1 – Key resources table. An unpaired two-samples *t* test was used for calculating the *P*-values, (ns *P* > 0.05, *P < 0.05, **P < 0.01, ***P < 0.001, ****P < 0.0001, exact values listed in Table S2), which correspond to the comparison between the control cell line and each NMD(+) reporter cell line for each siRNA. **(B)** Box plots showing the comparison between NMD(+) reporter mRNA and protein levels relative to control reporter levels, with and without NMD factor depletion (indicated on x-axis). Fold changes are shown for the difference between mRNA and protein levels under control conditions (∼4-fold), the difference between mRNA levels with and without SMG1 depletion (∼2.5-fold), and the difference between protein levels with and without SMG1 depletion (∼5-fold). An unpaired two-samples *t* test was used for calculating the *P*-values (**P < 0.01, ****P < 0.0001, exact values listed in Table S2), which correspond to the comparison between mRNA and protein levels under the same siRNA treatment conditions for each NMD(+) reporter cell line.

For more precise quantification of these changes, we used the dual-luciferase assay to measure protein levels in the NMD reporter cell lines and normalized to control siRNA conditions and the control cell line. The protein levels for both NMD(+) reporters increased to some degree with at least one siRNA for each targeted gene, with depletion of eIF4A3 and SMG1 leading to the biggest effect size and depletion of SMG6 showing a more modest effect size (Fig 3A). Surprisingly, the NMD(+) reporter protein levels did not increase substantially in both NMD(+) reporter cell lines treated with siRNAs targeting *UPF1* (Fig 3A), despite full depletion of the UPF1 protein (Fig S2, lanes 4–5). We plotted the data from the control siRNA conditions and normalized to just the control cell line to quantify the steady-state protein levels (Fig 3B). As expected, NMD(+) reporter protein levels were lower than control reporter levels under control siRNA conditions (Fig 3B, “Control” green boxes compared with “Control” black box).

**Figure S3.**
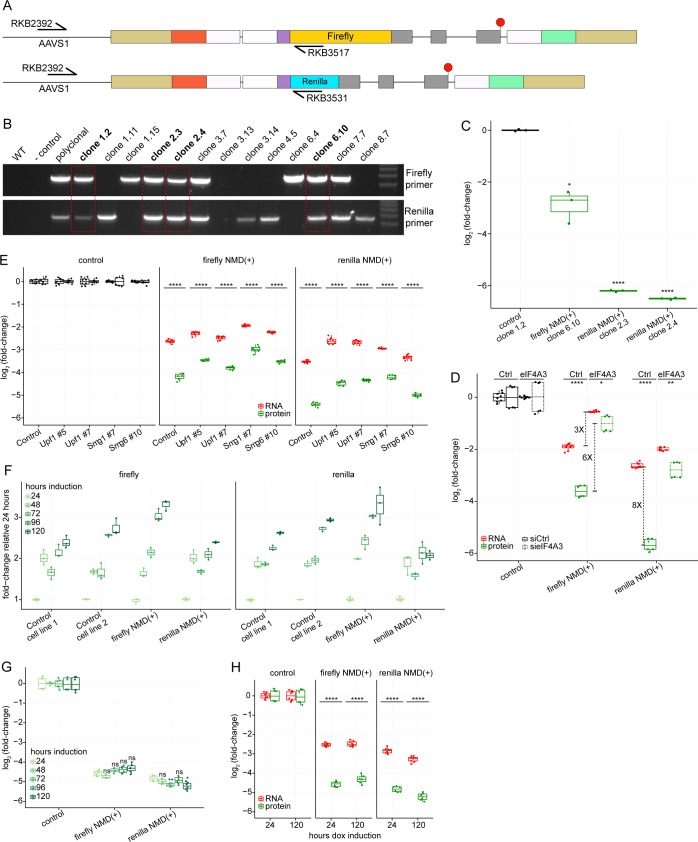
Quantification of NMD reporter mRNA and protein levels. **(A)** Schematic showing primer design to confirm integration of both reporters at the AAVS1 loci. The forward primer was designed upstream of the AAVS1 homology arm region, whereas the reverse primer was designed inside the luciferase sequence. The different primers for the different luciferase sequences allow us to distinguish whether the firefly and/or Renilla reporter is stably integrated. Primer sequences are listed in Table S1 – Key resources table. **(A, B)** Genomic DNA PCR using the primer sets shown in (A) showing which monoclonal cell lines have both luciferase reporters integrated at the AAVS1 loci. The bands outlined in the red boxes correspond to the clonal cell lines that had stable integration of both luciferase reporters at the AAVS1 loci and were subsequently used for dual-luciferase assay measurements. **(C)** Box plots showing the NMD reporter protein levels from monoclonal cell lines with both firefly and Renilla luciferase reporters stably integrated in the AAVS1 loci. The levels are normalized to the control reporter monoclonal cell line. Each box plot shows n = 3 technical replicates normalized to n = 1 biological replicate. An unpaired two-samples *t* test was used for calculating the *P*-values (*P < 0.05, ****P < 0.0001, exact values listed in Table S2), which correspond to the comparison between the monoclonal control cell line and each monoclonal NMD(+) reporter cell line. **(D)** Box plots showing additional experimental data for the comparison between NMD(+) reporter mRNA and protein levels relative to control reporter levels, as in Fig 3B. Fold-changes are shown for the difference between mRNA and protein levels under control conditions (∼8-fold), the difference between mRNA levels with and without eIF4A3 depletion (∼3-fold), and the difference between protein levels with and without eIF4A3 depletion (∼6-fold). An unpaired two-samples *t* test was used for calculating the *P*-values (*P < 0.05, **P < 0.01, ****P < 0.0001, exact values listed in Table S2), which correspond to the comparison between mRNA and protein levels under the same siRNA treatment conditions for each NMD(+) reporter cell line. **(E)** Box plots showing the comparison between NMD(+) reporter mRNA and protein levels relative to control reporter levels, with and without NMD factor depletion (indicated on x-axis). These data are plotted in the same manner as in Fig 3B, but the siRNAs used for these samples did not show as big of an effect size for the NMD(+) reporter RNA and protein levels as the siRNAs used for Fig 3B. An unpaired two-samples *t* test was used for calculating the *P*-values (****P < 0.0001, exact values listed in Table S2), which correspond to the comparison between mRNA and protein levels under the same siRNA treatment conditions for each NMD(+) reporter cell line. The siRNAs used for Fig 3B are Smg1 #5, Smg6 # 11, and eIF4A3, the details of which are in Table S1 – Key resources table. **(F)** Box plots showing the levels of both firefly (left plot) and Renilla (right plot) luminescence for each NMD reporter cell line for different lengths of time of reporter expression. The absolute luminescence levels were normalized to the levels from the 24-h dox induction time point for each luciferase in each cell line at each time point. Each box plot shows n = 3 technical replicates. **(G)** Box plots showing the NMD(+) reporter protein levels relative to control reporter protein levels for different lengths of time of reporter expression. Each box plot shows n = 3 technical replicates normalized to n = 2 biological replicates for a total of six data points. An unpaired two-samples *t* test was used for calculating the *P*-values (ns *P* > 0.05, *P < 0.05, **P < 0.01, exact values listed in Table S2), which correspond to the comparison of NMD(+) reporter protein levels at each time point relative to the 24-h dox induction time point. **(H)** Box plots showing the comparison between NMD(+) reporter mRNA and protein levels relative to control reporter levels for a short (24 h) and a long (120 h) length of time of reporter expression. For the protein levels, each box plot shows n = 3 technical replicates normalized to n = 2 biological replicates for a total of six data points. For the mRNA levels, each box plot shows n = 4 technical replicates normalized to n = 2 biological replicates for a total of eight data points. An unpaired two-samples *t* test was used for calculating the *P*-values (****P < 0.0001, exact values listed in Table S2) which correspond to the comparison between mRNA and protein levels for each dox induction time for each NMD(+) reporter cell line. Source data are available for this figure.

Although decreased NMD(+) reporter protein levels relative to control reporter levels were expected, the dramatic extent of this protein-level suppression was surprising. We therefore tested whether differential rates of integration of the reporters were responsible for these pronounced differences. We assessed whether this phenomenon was still observed in a more controlled genetic setting in which exactly one copy of both the control and NMD(+) reporter was stably integrated in every cell. We performed single-cell sorting, established monoclonal cell lines, and selected clones which we confirmed via gDNA PCR had both firefly and Renilla luciferase reporters stably integrated at the loci of the two AAVS1 alleles (Fig S3A and B, additional details in the Materials and Methods section). Monoclonal cell line protein levels mimicked those observed in the polyclonal lines (Fig S3C), confirming that biased integration was not the source of the marked protein-level suppression.

We next used the quantitative nature of our reporters to compare the relative suppression of mRNA and protein as a consequence of NMD. Unexpectedly, the NMD(+) reporter protein levels were consistently reduced to a greater degree than were the NMD(+) reporter mRNA levels (Fig 3B, green boxes compared to red boxes) relative to control reporters. For example, the Renilla NMD(+) reporter mRNA was reduced to ∼9% of control reporter mRNA levels, whereas the Renilla NMD(+) reporter protein was reduced to ∼2% of control reporter protein levels (Fig 3B). We repeated these measurements with an additional experiment and obtained similar results (Fig S3D). Overall, these experiments unexpectedly revealed four to eightfold greater suppression of protein levels than we observed with RNA levels.

We next tested whether this enhanced protein-level suppression arose from the NMD sensitivity of the reporter mRNA. We depleted multiple factors to inhibit NMD and measured mRNA and protein levels. Upon effective depletion of SMG1, SMG6, or eIF4A3, NMD(+) reporter protein levels increased relative to control siRNA conditions (Fig 3B, green boxes, additional data for less effective depletion in Fig S3E). NMD(+) reporter protein levels increased to a greater degree than did NMD(+) reporter mRNA levels with NMD inhibited (∼5-fold protein-level increase versus ∼2.5-fold mRNA-level increase following SMG1 depletion, annotated in Fig 3B). Furthermore, the NMD(+) reporter protein levels approached the same level as the NMD(+) reporter mRNA levels under SMG1 and eIF4A3 depletion conditions (green boxes versus red boxes), which is in stark contrast to the large differences under control siRNA conditions. These data imply that the pronounced difference in relative suppression of mRNA and protein levels is dependent on reporter NMD sensitivity.

A potential caveat to this phenomenon is that the reporter protein levels may not have reached steady state after the 24 h of doxycycline induced expression. To address this, we induced reporter expression at 24-h intervals up to 120 h. We observed that the reporter protein levels did continue to increase with longer expression (Fig S3F), but the normalized NMD(+) reporter protein levels relative to control reporter protein levels remained constant (Fig S3G). Furthermore, we measured reporter mRNA levels at a short (24 h) and long (120 h) length of expression time. We found that the NMD(+) reporter protein levels were reduced to a greater degree than the NMD(+) reporter mRNA levels for both short and long induction times (Fig S3H), confirming that the differences in relative suppression are not dependent on the length of time of expression of the reporters.

### NMD(+) reporter proteins are degraded modestly faster than are control reporter proteins

The greater degree of NMD(+) reporter protein reduction relative to mRNA reduction implies the existence of cellular mechanisms beyond RNA decay that limit the levels of proteins translated from NMD-sensitive transcripts. We therefore sought to test possible mechanisms responsible for this phenomenon.

**Figure 4.**
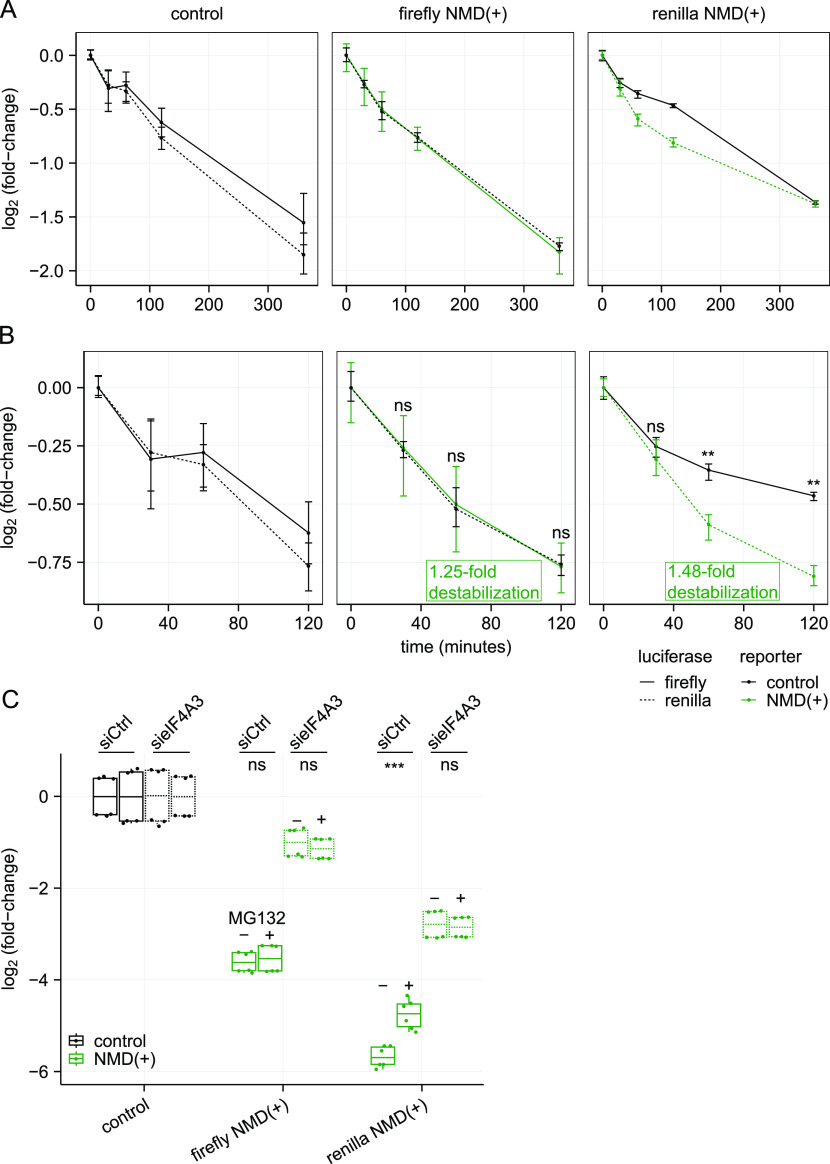
NMD(+) reporter proteins are degraded modestly faster than are control reporter proteins. **(A)** Line plots showing the decay kinetics of NMD reporter proteins after translation inhibition with cycloheximide. Each time point corresponds to n = 3 technical replicates (n = 2 biological replicates for the control panel, n = 6 data points), with error bars showing the range of those values and the line plot connecting at the mean of the values. **(B)** Same as in (A), but only for the early time points. *P*-values were calculated as described for Fig 2B (ns *P* > 0.05, **P < 0.01, exact values listed in Table S2), using the ratio of firefly:Renilla at each time point for the NMD(+) reporter cell lines compared with the control cell lines. The fold increase in destabilization/degradation of the NMD(+) reporter proteins relative to the corresponding control reporter proteins are based on the estimated half-lives of the reporter proteins calculated in Fig S4B. **(C)** Box plots showing NMD(+) reporter protein levels with and without MG132 treatment and eIF4A3 depletion relative to control reporter protein levels. An unpaired two-samples *t* test was used for calculating the *P*-values (ns *P* > 0.05, ***P < 0.001, exact values listed in Table S2).

One potential mechanism is through increased degradation of proteins encoded by NMD-sensitive mRNAs, which has been observed for truncated proteins encoded by NMD-sensitive reporter transcripts in yeast (Kuroha et al, 2009). To directly measure the decay kinetics of NMD reporter proteins, we inhibited translation with cycloheximide in our NMD reporter cell lines and measured protein levels at several later time points. Over the full 6-h time course, there was minimal change between control and NMD(+) protein levels (Fig 4A). It is possible that cycloheximide treatment could have led to unintended side effects in these cell lines, but we felt this was the best strategy for getting precise, quantitative measurements of the NMD(+) reporter proteins to estimate half-lives.

**Figure S4.**
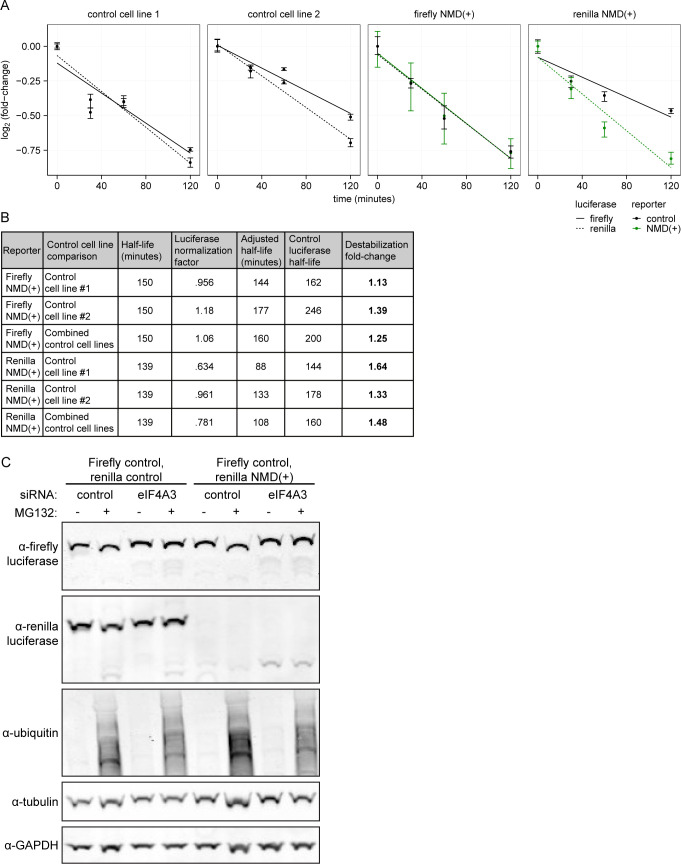
Estimating the half-lives of the NMD reporter proteins. **(A)** Same data as in Fig 4B, but with a best-fit line plotted as calculated from linear regression of the mean of the technical replicates at each time point for each reporter. The best fit line was used to calculate the unnormalized half-life for each reporter protein in each cell line based on the time (x-axis) at which the best-fit line crosses the 50% protein level (relative to time 0). The two control cell lines are plotted separately. **(B)** Table showing fold-change in destabilization of the NMD(+) reporter protein relative to the control reporter protein for each luciferase reporter. The half-lives of the reporter proteins in the NMD(+) cell lines were normalized to the half-lives of the reporter proteins in the control cell lines (using the half-lives of the control luciferase reporter proteins that both cell lines have in common) to more accurately estimate the differences in half-lives. The half-life of the control luciferase in the control reporter cell line was divided by the half-life of the corresponding control luciferase in the NMD(+) reporter cell line (e.g., control reporter cell line firefly/NMD(+) reporter cell line firefly) to get the “luciferase normalization factor.” The half-life of the NMD(+) luciferase in the NMD(+) reporter cell line was then multiplied by the luciferase normalization factor to get the “adjusted half-life.” The half-life of the corresponding control luciferase in the control reporter cell line was then divided by the adjusted half-life of the NMD(+) luciferase in the NMD(+) reporter cell line (e.g., control reporter cell line Renilla half-life/NMD(+) reporter cell line Renilla adjusted half-life) to get the fold-change in destabilization of the NMD(+) reporter protein relative to the control reporter protein. For each NMD(+) reporter luciferase, the comparison was made with each control cell line separately and the two control cell lines combined (“Control cell line comparison” column). **(C)** Western blots confirming that MG132 treatment led to proteasomal inhibition and accumulation of ubiquitinated proteins. The polyclonal NMD reporter cell line used for each sample is indicated above the blot. Samples for this blot are from cells lysed in passive lysis buffer (PLB) for use in the Dual-Luciferase Reporter Assay. Source data are available for this figure.

Interestingly, the early time points do show faster degradation of NMD(+) reporter proteins relative to control reporter proteins (Fig 4B). However, the changes are relatively modest. To estimate the differences in half-lives of the reporter proteins at these early time points, we estimated best-fit exponential decay models (Fig S4A) and normalized to the control cell lines. Although the amino acid sequence differences between the firefly and Renilla proteins could potentially differentially affect protein stability, this is not a confounding factor in our measurements because we normalized all data for proteins produced from NMD(+) transcripts to corresponding data for the firefly and Renilla control proteins produced from NMD-insensitive transcripts. Our measurements are therefore internally controlled for amino acid sequence. The NMD(+) reporter proteins were degraded ∼1.1–1.6-fold faster than were the control reporter proteins (Fig S4B), a modest change similar in magnitude to that of a protein from an NMD-targeted transcript in a recent report using a different reporter system (Chu et al, 2021). In contrast, steady-state protein levels were ∼4- to 8-fold lower than were steady-state mRNA levels (Fig 3B), suggesting that increased degradation of proteins encoded by NMD-sensitive transcripts is not the primary mechanism underlying the marked protein-level suppression that we observed.

Table S1 Key resources table.

Table S2 Statistical significance values.

Table S3 Median box plot values as percentages.

We next sought to determine if the modest increase in NMD(+) reporter protein decay that we observed was dependent on the ubiquitin-proteasome system. We treated our cell lines with MG132 to inhibit the proteasome and measured reporter protein levels. We confirmed that MG132 treatment was functional by confirming an increase in global protein ubiquitination (Fig S4C). We observed no or very modest increases in NMD(+) reporter protein levels with MG132 treatment relative to no MG132 (Fig 4C, solid green boxes), consistent with modest increases in degradation rate (Fig 4B). Although the effects of MG132 treatment on protein levels were more notable for the Renilla NMD(+) reporter than the firefly NMD(+) reporter, the effects for both were dwarfed by the effects of eIF4A3 depletion on protein levels (Fig 4C, dashed green boxes). Together, these data demonstrate that increased protein degradation is not the dominant mechanism leading to lower observed steady-state levels.

## Discussion

We have developed a robust NMD reporter system for making precise, quantitative mRNA and protein level measurements (Fig 1). This system builds on previous reporters and adds numerous features, including (1) luciferase domains for high dynamic range protein-level measurements, (2) internal control reporters for accurate normalization across samples, (3) dox inducibility for mRNA stability measurements, and (4) stable integration at the AAVS1 safe harbor loci for predictable genomic integration and uniform expression. The highly controlled nature of these reporters permitted us to clearly demonstrate that protein levels of the NMD(+) reporters were reduced to a greater degree than were mRNA levels, and to quantify the relative magnitude of mRNA- and protein-level suppression (Fig 3). Together with previous studies reporting protein-level suppression in both yeast and human cells, these findings imply that cells use mechanisms beyond mRNA decay to reduce the levels of potentially deleterious truncated proteins encoded by NMD-sensitive transcripts.

The modest increase in decay of the NMD(+) reporter proteins at early time points (Fig 4B) suggests that cells may have a mechanism to target truncated proteins for degradation, similar to the ribosome-associated protein quality control (RQC) pathway (Joazeiro, 2019). However, similar control and NMD(+) reporter protein levels at the late time point (Fig 4A) appear inconsistent with the existence of such a mechanism. One way to reconcile these differences is to hypothesize the presence of two populations of NMD transcripts: one consisting of transcripts that are rapidly degraded, and the other with transcripts that “escape” NMD and are degraded at a similar rate as control transcripts; only proteins derived from rapidly degraded transcripts are rapidly degraded themselves. This hypothesis is consistent with previous studies demonstrating the existence of two such pools of NMD-sensitive transcripts (Cheng & Maquat, 1993; Belgrader et al, 1994; Trcek et al, 2013; Kim et al, 2017; Hoek et al, 2019). However, such a mechanism still would not fully explain the large difference between NMD(+) reporter mRNA and protein steady-state levels (Fig 3B), suggesting that additional mechanisms, such as reduced translation, likely modulate protein levels. Reduced translation of NMD-sensitive mRNAs has been observed in previous studies (Ishigaki et al, 2001; Chiu et al, 2004; Sheth & Parker, 2006; You et al, 2007; Isken et al, 2008; Lee et al, 2010; Kim et al, 2017) and a recent report identified factors involved in translational repression specifically of NMD-sensitive transcripts (Zinshteyn et al, 2021). Given the minimal protein decay differences that we observed (Fig 4B), translational repression is likely an important mechanism for limiting levels of proteins encoded by NMD-sensitive transcripts.

**Figure 5.**
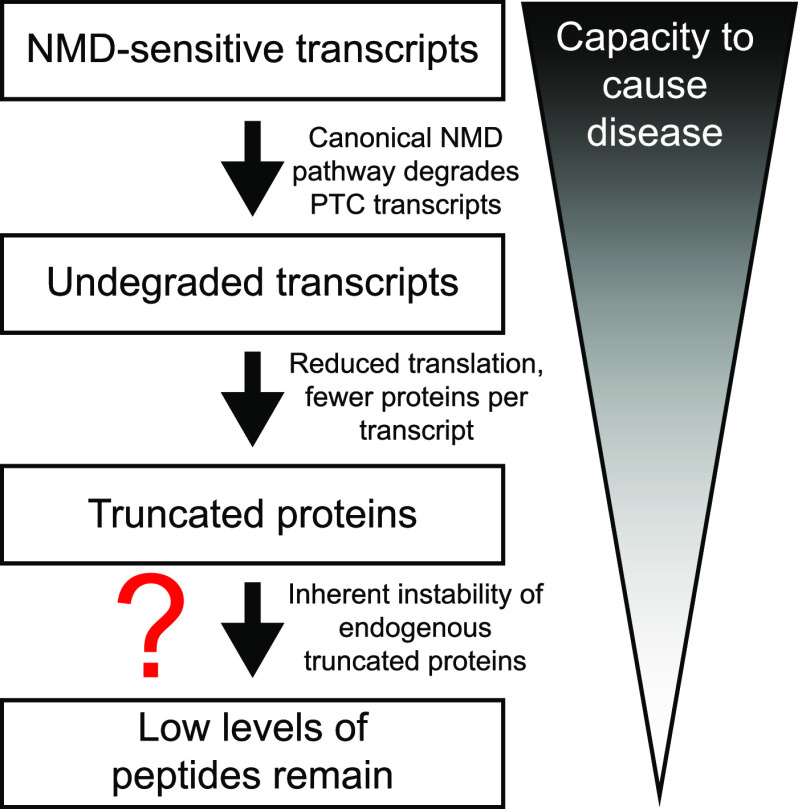
Model illustrating multiple layers of NMD pathway that complement one another to limit truncated protein accumulation.

Our data suggest a model in which there are multiple layers of the NMD pathway, each of which acts to limit truncated protein accumulation (Fig 5). The first is the canonical, well-characterized mRNA degradation pathway, preventing truncated protein production by reducing the NMD-sensitive mRNA available to make proteins. The second is through limiting the accumulation of truncated proteins from the remaining mRNAs (via modestly increased protein degradation and reduced translation), leading to protein levels at a fraction of those of full-length proteins translated from NMD-insensitive transcripts. Neither mechanism is 100% efficient (some mRNAs escape NMD and some proteins are still translated from the remaining mRNAs), but the combination of both leads to an additive decrease in protein accumulation that prevents deleterious effects on cell health. A potential additional layer of modulation for truncated peptides encoded by endogenous NMD-sensitive mRNAs is inherent instability, which could lead to substantially faster degradation and even lower protein levels.

The presumed purpose of NMD is to limit the accumulation of truncated proteins that could negatively affect cell health and homeostasis. The canonical mechanism for this is through recognition and degradation of NMD-sensitive mRNA, which is the well-characterized NMD pathway. Our data provide evidence for mechanisms complementary to the canonical pathway that further act to prevent the accumulation of truncated proteins. Future work is needed to determine if the limited protein accumulation from these NMD reporters is representative of most or all NMD-sensitive transcripts and their encoded proteins, and how other features—of both the transcript and peptide sequence—can influence this phenomenon.

## Materials and Methods

### Design and cloning of luciferase-based NMD reporters

A set of firefly luciferase NMD reporters were obtained as a gift from Dr. James Inglese (Addgene IDs: 112085 and 112084). These reporters had the firefly luciferase sequence followed by either full-length or PTC39 β-globin sequence in the p3xFLAG-CMV-10 backbone (Baird et al, 2018). To create Renilla luciferase versions of these reporters, the plasmids were digested with EcoRI to cut on either side of the firefly luciferase sequence. The Renilla luciferase sequence was amplified via PCR using two sets of primers with overhang sequences to facilitate isothermal assembly into the cut backbone (primer sequences listed in Table S1 – Key resources table, RKB3257-3260). The Renilla luciferase PCR amplicon (insert) and cut backbone were run on a 1% agarose gel and the DNA was isolated by gel extraction. The insert was then ligated into the cut backbone by isothermal assembly (NEBuilder HiFi DNA Assembly, E2621L) according to the manufacturer’s protocol. The assembled plasmids were transformed into NEB Stable Competent *Escherichia coli* (C3040H) and individual colonies were sequence verified.

The transient expression constructs described above were sub-cloned into donor plasmid backbones for CRISPR/Cas9-mediated genomic integration via homology directed repair. A backbone with homology arms to the AAVS1 safe harbor locus and with a dox-inducible promoter (Natsume et al, 2016; Addgene ID 72835) was cut with MluI and BglII. The luciferase-β-globin sequence was amplified via PCR using primers with overhang sequences (RKB3454-3455) to facilitate isothermal assembly into the cut backbone. The cloning proceeded as described above, with the final assembled plasmids sequence verified. All of these plasmids are available on Addgene (see Table S1 – Key resources table).

### Cell culture and genome engineering of HEK 293 cells

Flp-In T-REx 293 cells (R78007; Thermo Fisher Scientific) were cultured at 37°C and 5% CO_2_ in DMEM media with 10% FBS and 1% penicillin–streptomycin and split every 2–3 d before reaching full confluence. For stable integration of the NMD reporters, 293 cells were plated at 40% confluency in a well of a 12-well plate the day before transfecting. For each well, ∼400 ng of firefly donor plasmid, ∼400 ng of Renilla donor plasmid, and ∼400 ng of Cas9/AAVS1-sgRNA expressing plasmid were used. The DNA and 2.4 μl of P3000 reagent were diluted in 60 μl of Opti-MEM (Gibco) and separately 1.8 μl of Lipofectamine 3000 Reagent (L3000-015; Invitrogen) was diluted in 60 μl Opti-MEM. The transfections proceeded according to the manufacturer’s protocol, with 100 μl of transfection mix added to each well already containing 1 ml of medium.

1 d after transfecting, puromycin (A11138-03; Gibco) was added (2 μg/ml) to select for successfully transfected cells. The next day, cells were split from each well into a 10-cm plate and grown in puromycin-containing medium for several days to select for stable integration of the reporters. Polyclonal cell lines were cryopreserved and used for subsequent experiments.

### Induction of reporters and depletion of NMD factors

For RNAi-mediated knockdown of NMD factors, reverse transfections were used to add siRNAs to our cells. For each reverse transfection, 3 μl of Lipofectamine RNAiMAX Reagent (13778-150; Invitrogen) was diluted in 50 μl of Opti-MEM (31985-062; Gibco) and separately siRNA was diluted in 50 μl of Opti-MEM. These volumes were combined, mixed, and incubated at room temperature for 10 min. 100 μl of the transfection mix was added to the bottom of an empty well of a 12-well culture plate. Cells resuspended in 900 μl of medium were plated into each well at ∼20% confluence, aiming for ∼100% confluence 72 h after plating. The final concentration of siRNA for each well was 20 nM (4 μl of 5 μM siRNA for these transfections) in 1 ml final volume per well. The cells were collected 72 h after transfection. To induce expression of the stably integrated reporters, doxycycline (D9891; Sigma-Aldrich) was added to a final concentration of 1 μg/ml to each well 24 h before cell collection. For inducing reporter expression for multiple lengths of time, the cells were plated in five separate 12-well plates and doxycycline was added at a different time (24-h intervals) for each plate. Doxycycline was replenished every 24 h and cells passaged to new plates every 48 h.

### RNA extraction and RT-PCR to confirm expected splicing of reporter mRNA

Cell pellets were lysed with TRIzol Reagent (15596-026; Invitrogen) and processed according to the manufacturer’s protocol for RNA isolation. The RNA pellet was resuspended in 50 μl of water and then subjected to RNeasy (74104; QIAGEN) column purification and DNase digestion. The 50 μl of RNA was added to 300 μl RLT lysis buffer, mixed with 350 μl 70% ethanol, and transferred to an RNeasy spin column. The samples were then processed according to the manufacturer’s protocol using RNase-Free DNase Set (79254; QIAGEN) for on-column DNase digestion.

The purified RNA was confirmed to be free of DNA contamination using end point PCR with primers specific to the firefly luciferase sequence (RKB2250-2251). cDNA was synthesized from 3 μg of RNA per sample using SuperScript IV Reverse Transcriptase (18090050; Invitrogen) using oligo-dT primers according to the manufacturer’s protocol. To check splicing, forward primers were designed in either the firefly or Renilla sequence and reverse primers were designed in the 3′-UTR sequence just downstream of the last β-globin exon. End-point RT-PCR was performed on the cDNA samples using these primers (RKB3600-3612, Fig S1B and C), and the PCRs were run on 1% agarose gels to visualize the size of the PCR amplicons.

### NMD reporter mRNA steady-state level measurement

The cDNA reactions were diluted 1:50 and 4 μl was used per 10 μl qRT-PCR reaction with PowerUp SYBR Green Master Mix (A25742; Thermo Fisher Scientific) and final primer concentrations of 500 nM in 384-well plates (AB1384; Thermo Fisher Scientific). Two unique primer sets (Table S1 – Key resources table) were used for each luciferase and two reference genes were also quantified with qRT-PCR using an ABI QuantStudio 5 Real-Time PCR System (Thermo Fisher Scientific). Three technical replicate reactions were performed for each unique primer set for each sample.

To quantify the reporter mRNA levels from the qRT-PCR data, the means of the technical replicates for each reporter primer set were used. Each of the two firefly primer sets was compared with each of the two Renilla primer sets, for a total of n = 4 normalized technical replicates in each sample. Those replicates were normalized to replicates from two control cell lines for a total of eight data points, all plotted relative to the control cell lines.

### Decay kinetics of NMD reporter mRNA

Cells were plated at 10% confluency in poly-L-lysine coated wells of a 12-well culture plate the day before induction. The following day, the medium was replaced with doxycycline-containing medium 24 h before cell collection. To turn off reporter expression to measure mRNA decay kinetics, doxycycline-containing medium was removed and the cells were washed with PBS and standard medium (no dox) before being replaced with the standard medium for a specified length of time before the cells were harvested. RNA extraction, cDNA synthesis, and qRT-PCR were performed as described above.

For examining reporter mRNA decay kinetics from qRT-PCR data, each of the luciferase primer sets (two sets for firefly and two sets for Renilla) was normalized to two reference genes (*RPL27* and *SRP14*) for a total of n = 4 technical replicates for each luciferase in each sample. Those values were normalized to the sample collected at time 0 for each cell line and plotted relative to time 0 to show the decrease in reporter mRNA over time.

### Western blotting for NMD reporter proteins

Cells were collected from individual wells of a 12-well culture plate, centrifuged at 200*g* for 5 min, washed with PBS, and lysed using 50 μl of NP40 Cell Lysis Buffer (FNN0021; Invitrogen) supplemented with protease and phosphatase inhibitors (A32955; Pierce). Lysates were sonicated, incubated on ice, and spun down at 10,000*g* at 4°C for 15 min; the supernatant was collected for downstream assays. The protein concentration was quantified using the Qubit Protein Assay (Q33212; Thermo Fisher Scientific). 12 μg of protein per sample was used for gel electrophoresis with a NuPAGE 4–12% Mini Protein Gel (NP0323; Invitrogen) in a Mini Gel Tank (Invitrogen) with 1X NuPAGE MOPS SDS Running Buffer (NP0001; Invitrogen). After electrophoresis, protein was transferred overnight to a nitrocellulose membrane (LC2001; Invitrogen) using 1X NuPAGE Transfer Buffer (NP0006-1; Invitrogen) with 10% methanol. After transfer, protein bands were visualized with Ponceau stain (P7170; Sigma-Aldrich) to confirm protein transfer and even loading.

The membrane was blocked with Odyssey Blocking Buffer (PBS) (927-40000; LI-COR Biosciences) for 1 h at 4°C with gentle shaking. The blot was probed with primary antibodies overnight at 4°C with gentle shaking; specific antibodies and dilutions used are listed in Table S1 – Key resources table. After overnight incubation, the blot was washed three times for 5 min with 1X TBST buffer at room temperature with gentle shaking. The blot was then probed with IRDye secondary antibodies (LI-COR Biosciences) for 1 h at room temperature with gentle shaking, followed by a final sequence of washes as described above. The blot was imaged on an Odyssey CLx Imaging System and images were processed using Fiji (ImageJ v2.1.0).

The Western blot did not show full depletion of the eIF4A3 protein. However, the immunogen sequence used to generate the eIF4A3 antibody is highly conserved among eIF4A3, eIF4A1, and eIF4A2, and all three proteins are similar size. We predict that the antibody is likely also binding eIF4A1 and eIF4A2 on the Western blot and the band shown corresponds to all three of those proteins. Given that the bands have modestly reduced intensity and the NMD reporter protein levels increase in the lanes with sieIF4A3 samples, we concluded that the siRNAs are likely effectively depleting eIF4A3.

### Dual-luciferase assay for NMD reporter protein level measurement

The Dual-Luciferase Reporter Assay System (E1910; Promega) was used for measuring luciferase levels from NMD reporter cell lines according to the manufacturer’s protocol. Briefly, individual wells of a 12-well culture plate were washed with 1 ml of PBS before 200 μl of 1X Passive Lysis Buffer was added directly to each well. The culture plates were placed on an orbital shaker with gentle rocking for 15 min at room temperature to achieve complete lysis. Cell lysates were collected and centrifuged at max speed for 30 s and the supernatants were collected and used for subsequent assays.

The dual-luciferase assay was performed using a Cytation 5 plate reader with a dual-injector system (BioTek). For each sample, 20 μl of lysate was transferred to wells of a 96-well plate. The plate reader was set-up and programmed to inject 100 μl of Luciferase Assay Reagent II (LAR II) from the first injector and 100 μl of Stop & Glo Reagent from the second injector. Timing for measuring luminescence was set according to the Dual-Luciferase Reporter Assay System protocol.

### Monoclonal NMD reporter cell line generation

Polyclonal cell lines underwent single cell sorting into 96-well culture plates using an MA900 multi-application cell sorter (Sony Biotechnology). Single cells were grown in 50 μl of DMEM medium supplemented with 20% FBS per well, with an additional 50 μl of medium added every 3–4 d to maintain optimal growth conditions for cells at low confluence. Cells began reaching confluence in individual wells 2–3 wk after sorting, at which time the cells were split into 24-well plates. Upon reaching confluence in the 24-well plates, cells were split into two separate 12-well plates: one to continue propagating cells and another for a dual-luciferase assay to determine which luciferase reporters were stably integrated. Monoclonal lines with luciferase expression were cryo-preserved and a subset of cells from each line were collected for gDNA extraction with DNeasy Blood & Tissue Kit (69504; QIAGEN) to confirm stable integration of the reporters at the AAVS1 loci via gDNA-PCR.

A forward primer was designed in the AAVS1 sequence outside of the left homology arm on the donor plasmid (RKB2392), whereas the reverse primer was designed in the luciferase sequence (Fig S3A) such that only stable integration of the reporters at that locus would yield a PCR amplicon. Separate reverse primers were designed for firefly and Renilla luciferase sequences (RKB3517 and RKB3531), and monoclonal lines with amplicons specific to both luciferases were used for subsequent experiments.

### Proteasome inhibition in NMD reporter cell lines

Reporter cell lines were grown in 12-well culture dishes and treated with siRNAs as described above. Cells were treated with a final concentration of 10 μM MG132 (C2211; Sigma-Aldrich) to inhibit the proteasome; control samples were treated with DMSO (vehicle) (D2650; Sigma-Aldrich). Cells were lysed in 200 μl of 1X Passive Lysis Buffer (dual-luciferase assay) 6 h after MG132 addition. The samples were processed for use in the dual-luciferase assay as described above.

### Decay kinetics of NMD reporter proteins

Cells were grown in 12-well culture plates and reporter expression was induced with doxycycline as described above. To inhibit translation, cells were treated with cycloheximide (C7698; Sigma-Aldrich) at a final concentration of 100 μg/ml for specified lengths of time before being lysed with 200 μl of 1X Passive Lysis Buffer. The samples were processed for use in the dual-luciferase assay.

For examining reporter protein decay kinetics from the dual-luciferase assay data, the luminescence values for each luciferase were plotted relative to the time 0 value to show the change in reporter protein levels over time after translation inhibition. The half-lives of the reporters were calculated using linear regression of the mean of the technical replicates at each time point for each reporter. The half-lives of the NMD(+) reporter proteins were normalized to those of the control reporter proteins and control reporter cell line to get the “fold destabilization” (Fig 4B) relative to the control reporter.

## Data Availability

All source data are available as source data files.

## Supplementary Material

Reviewer comments

## References

[bib1] Aksit MA, Bowling AD, Evans TA, Joynt AT, Osorio D, Patel S, West N, Merlo C, Sosnay PR, Cutting GR, (2019) Decreased mRNA and protein stability of W1282X limits response to modulator therapy. J Cyst Fibros 18: 606–613. 10.1016/j.jcf.2019.02.009 30803905 PMC6706327

[bib2] Alexandrov A, Shu MD, Steitz JA (2017) Fluorescence amplification method for forward genetic discovery of factors in human mRNA degradation. Mol Cell 65: 191–201. 10.1016/j.molcel.2016.11.032 28017590 PMC5301997

[bib3] Anczuków O, Ware MD, Buisson M, Zetoune AB, Stoppa-Lyonnet D, Sinilnikova OM, Mazoyer S (2008) Does the nonsense-mediated mRNA decay mechanism prevent the synthesis of truncated BRCA1, CHK2, and p53 proteins? Hum Mutat 29: 65–73. 10.1002/humu.20590 17694537

[bib4] Andersen CB, Ballut L, Johansen JS, Chamieh H, Nielsen KH, Oliveira CL, Pedersen JS, Séraphin B, Le Hir H, Andersen GR (2006) Structure of the exon junction core complex with a trapped DEAD-box ATPase bound to RNA. Science 313: 1968–1972. 10.1126/science.1131981 16931718

[bib5] Baird TD, Cheng KC, Chen YC, Buehler E, Martin SE, Inglese J, Hogg JR (2018) ICE1 promotes the link between splicing and nonsense-mediated mRNA decay. Elife 7: e33178. 10.7554/eLife.33178 29528287 PMC5896957

[bib6] Belgrader P, Cheng J, Zhou X, Stephenson LS, Maquat LE (1994) Mammalian nonsense codons can be cis effectors of nuclear mRNA half-life. Mol Cell Biol 14: 8219–8228. 10.1128/mcb.14.12.8219 7969159 PMC359361

[bib7] Boelz S, Neu-Yilik G, Gehring NH, Hentze MW, Kulozik AE (2006) A chemiluminescence-based reporter system to monitor nonsense-mediated mRNA decay. Biochem Biophys Res Commun 349: 186–191. 10.1016/j.bbrc.2006.08.017 16934750

[bib8] Bono F, Ebert J, Lorentzen E, Conti E (2006) The crystal structure of the exon junction complex reveals how it maintains a stable grip on mRNA. Cell 126: 713–725. 10.1016/j.cell.2006.08.006 16923391

[bib9] Bühler M, Paillusson A, Mühlemann O (2004) Efficient downregulation of immunoglobulin mu mRNA with premature translation-termination codons requires the 5′-half of the VDJ exon. Nucleic Acids Res 32: 3304–3315. 10.1093/nar/gkh651 15210863 PMC443527

[bib10] Carter MS, Li S, Wilkinson MF (1996) A splicing-dependent regulatory mechanism that detects translation signals. EMBO J 15: 5965–5975. 10.1002/j.1460-2075.1996.tb00983.x 8918474 PMC452383

[bib11] Celik A, Baker R, He F, Jacobson A (2017) High-resolution profiling of NMD targets in yeast reveals translational fidelity as a basis for substrate selection. RNA 23: 735–748. 10.1261/rna.060541.116 28209632 PMC5393182

[bib12] Chan CC, Dostie J, Diem MD, Feng W, Mann M, Rappsilber J, Dreyfuss G (2004) eIF4A3 is a novel component of the exon junction complex. RNA 10: 200–209. 10.1261/rna.5230104 14730019 PMC1370532

[bib13] Chang JC, Temple GF, Trecartin RF, Kan YW (1979) Suppression of the nonsense mutation, mutation in homozygous beta 0 thalassaemia. Nature 281: 602–603. 10.1038/281602a0 492326

[bib14] Cheng J, Maquat LE (1993) Nonsense codons can reduce the abundance of nuclear mRNA without affecting the abundance of pre-mRNA or the half-life of cytoplasmic mRNA. Mol Cell Biol 13: 1892–1902. 10.1128/mcb.13.3.1892 8441420 PMC359503

[bib15] Cheruiyot A, Li S, Srivatsan SN, Ahmed T, Chen Y, Lemacon DS, Li Y, Yang Z, Wadugu BA, Warner WA, (2021) Nonsense-mediated RNA decay is a unique vulnerability of cancer cells harboring SF3B1 or U2AF1 mutations. Cancer Res 81: 4499–4513. 10.1158/0008-5472.CAN-20-4016 34215620 PMC8416940

[bib16] Chiu SY, Lejeune F, Ranganathan AC, Maquat LE (2004) The pioneer translation initiation complex is functionally distinct from but structurally overlaps with the steady-state translation initiation complex. Genes Dev 18: 745–754. 10.1101/gad.1170204 15059963 PMC387415

[bib17] Chu V, Feng Q, Lim Y, Shao S (2021) Selective destabilization of polypeptides synthesized from NMD-targeted transcripts. Mol Biol Cell 32: ar38. 10.1091/mbc.E21-08-0382 34586879 PMC8694075

[bib18] Coban-Akdemir Z, White JJ, Song X, Jhangiani SN, Fatih JM, Gambin T, Bayram Y, Chinn IK, Karaca E, Punetha J, (2018) Identifying genes whose mutant transcripts cause dominant disease traits by potential gain-of-function alleles. Am J Hum Genet 103: 171–187. 10.1016/j.ajhg.2018.06.009 30032986 PMC6081281

[bib19] Colombo M, Karousis ED, Bourquin J, Bruggmann R, Mühlemann O (2017) Transcriptome-wide identification of NMD-targeted human mRNAs reveals extensive redundancy between SMG6- and SMG7-mediated degradation pathways. RNA 23: 189–201. 10.1261/rna.059055.116 27864472 PMC5238794

[bib20] Daar IO, Maquat LE (1988) Premature translation termination mediates triosephosphate isomerase mRNA degradation. Mol Cell Biol 8: 802–813. 10.1128/mcb.8.2.802 2832737 PMC363207

[bib21] D’Orazio KN, Lessen LN, Veltri AJ, Neiman Z, Pacheco M, Loll-Krippleber R, Brown GW, Green R (2021) Genetic screens identify connections between ribosome recycling and nonsense mediated decay. BioRxiv. 10.1101/2021.08.03.454884. (Preprint posted August 03, 2021). PMC10917514 38198768

[bib22] Durand S, Lykke-Andersen J (2013) Nonsense-mediated mRNA decay occurs during eIF4F-dependent translation in human cells. Nat Struct Mol Biol 20: 702–709. 10.1038/nsmb.2575 23665580

[bib23] Eberle AB, Lykke-Andersen S, Mühlemann O, Jensen TH (2009) SMG6 promotes endonucleolytic cleavage of nonsense mRNA in human cells. Nat Struct Mol Biol 16: 49–55. 10.1038/nsmb.1530 19060897

[bib24] Eberle AB, Stalder L, Mathys H, Orozco RZ, Mühlemann O (2008) Posttranscriptional gene regulation by spatial rearrangement of the 3′ untranslated region. PLoS Biol 6: e92. 10.1371/journal.pbio.0060092 18447580 PMC2689704

[bib25] Ferraiuolo MA, Lee CS, Ler LW, Hsu JL, Costa-Mattioli M, Luo MJ, Reed R, Sonenberg N (2004) A nuclear translation-like factor eIF4AIII is recruited to the mRNA during splicing and functions in nonsense-mediated decay. Proc Natl Acad Sci U S A 101: 4118–4123. 10.1073/pnas.0400933101 15024115 PMC384704

[bib26] Gerbracht JV, Boehm V, Gehring NH (2017) Plasmid transfection influences the readout of nonsense-mediated mRNA decay reporter assays in human cells. Sci Rep 7: 10616. 10.1038/s41598-017-10847-4 28878343 PMC5587671

[bib27] Giorgi C, Yeo GW, Stone ME, Katz DB, Burge C, Turrigiano G, Moore MJ (2007) The EJC factor eIF4AIII modulates synaptic strength and neuronal protein expression. Cell 130: 179–191. 10.1016/j.cell.2007.05.028 17632064

[bib28] Gossen M, Freundlieb S, Bender G, Müller G, Hillen W, Bujard H (1995) Transcriptional activation by tetracyclines in mammalian cells. Science 268: 1766–1769. 10.1126/science.7792603 7792603

[bib29] Heinz N, Schambach A, Galla M, Maetzig T, Baum C, Loew R, Schiedlmeier B (2011) Retroviral and transposon-based tet-regulated all-in-one vectors with reduced background expression and improved dynamic range. Hum Gene Ther 22: 166–176. 10.1089/hum.2010.099 20825282

[bib30] Hoek TA, Khuperkar D, Lindeboom R, Sonneveld S, Verhagen B, Boersma S, Vermeulen M, Tanenbaum ME (2019) Single-molecule imaging uncovers rules governing nonsense-mediated mRNA decay. Mol Cell 75: 324–339.e11. 10.1016/j.molcel.2019.05.008 31155380 PMC6675935

[bib31] Holbrook JA, Neu-Yilik G, Hentze MW, Kulozik AE (2004) Nonsense-mediated decay approaches the clinic. Nat Genet 36: 801–808. 10.1038/ng1403 15284851

[bib32] Huntzinger E, Kashima I, Fauser M, Saulière J, Izaurralde E (2008) SMG6 is the catalytic endonuclease that cleaves mRNAs containing nonsense codons in metazoan. RNA 14: 2609–2617. 10.1261/rna.1386208 18974281 PMC2590965

[bib33] Inoue K, Khajavi M, Ohyama T, Hirabayashi S, Wilson J, Reggin JD, Mancias P, Butler IJ, Wilkinson MF, Wegner M, (2004) Molecular mechanism for distinct neurological phenotypes conveyed by allelic truncating mutations. Nat Genet 36: 361–369. 10.1038/ng1322 15004559

[bib34] Ishigaki Y, Li X, Serin G, Maquat LE (2001) Evidence for a pioneer round of mRNA translation: mRNAs subject to nonsense-mediated decay in mammalian cells are bound by CBP80 and CBP20. Cell 106: 607–617. 10.1016/s0092-8674(01)00475-5 11551508

[bib35] Isken O, Kim YK, Hosoda N, Mayeur GL, Hershey JW, Maquat LE (2008) Upf1 phosphorylation triggers translational repression during nonsense-mediated mRNA decay. Cell 133: 314–327. 10.1016/j.cell.2008.02.030 18423202 PMC4193665

[bib36] Joazeiro C (2019) Mechanisms and functions of ribosome-associated protein quality control. Nat Rev Mol Cell Biol 20: 368–383. 10.1038/s41580-019-0118-2 30940912 PMC7138134

[bib37] Kang JQ, Shen W, Macdonald RL (2009) Two molecular pathways (NMD and ERAD) contribute to a genetic epilepsy associated with the GABA(A) receptor GABRA1 PTC mutation, 975delC, S326fs328X. J Neurosci 29: 2833–2844. 10.1523/JNEUROSCI.4512-08.2009 19261879 PMC2687144

[bib38] Karousis ED, Gypas F, Zavolan M, Mühlemann O (2021) Nanopore sequencing reveals endogenous NMD-targeted isoforms in human cells. Genome Biol 22: 223. 10.1101/2021.04.30.442116 34389041 PMC8361881

[bib39] Khajavi M, Inoue K, Lupski JR (2006) Nonsense-mediated mRNA decay modulates clinical outcome of genetic disease. Eur J Hum Genet 14: 1074–1081. 10.1038/sj.ejhg.5201649 16757948

[bib40] Kim WK, Yun S, Kwon Y, You KT, Shin N, Kim J, Kim H (2017) mRNAs containing NMD-competent premature termination codons are stabilized and translated under UPF1 depletion. Sci Rep 7: 15833. 10.1038/s41598-017-16177-9 29158530 PMC5696521

[bib41] Kinniburgh AJ, Maquat LE, Schedl T, Rachmilewitz E, Ross J (1982) mRNA-deficient beta o-thalassemia results from a single nucleotide deletion. Nucleic Acids Res 10: 5421–5427. 10.1093/nar/10.18.5421 6292840 PMC320886

[bib42] Kovalak C, Donovan S, Bicknell AA, Metkar M, Moore MJ (2021) Deep sequencing of pre-translational mRNPs reveals hidden flux through evolutionarily conserved alternative splicing nonsense-mediated decay pathways. Genome Biol 22: 132. 10.1186/s13059-021-02309-y 33941243 PMC8091538

[bib43] Kuroha K, Tatematsu T, Inada T (2009) Upf1 stimulates degradation of the product derived from aberrant messenger RNA containing a specific nonsense mutation by the proteasome. EMBO Rep 10: 1265–1271. 10.1038/embor.2009.200 19798102 PMC2775173

[bib44] Kurosaki T, Miyoshi K, Myers JR, Maquat LE (2018) NMD-degradome sequencing reveals ribosome-bound intermediates with 3′-end non-templated nucleotides. Nat Struct Mol Biol 25: 940–950. 10.1038/s41594-018-0132-7 30275517 PMC8262411

[bib45] Kurosaki T, Popp MW, Maquat LE (2019) Quality and quantity control of gene expression by nonsense-mediated mRNA decay. Nat Rev Mol Cell Biol 20: 406–420. 10.1038/s41580-019-0126-2 30992545 PMC6855384

[bib46] Le Hir H, Gatfield D, Izaurralde E, Moore MJ (2001) The exon-exon junction complex provides a binding platform for factors involved in mRNA export and nonsense-mediated mRNA decay. EMBO J 20: 4987–4997. 10.1093/emboj/20.17.4987 11532962 PMC125616

[bib47] Le Hir H, Izaurralde E, Maquat LE, Moore MJ (2000) The spliceosome deposits multiple proteins 20-24 nucleotides upstream of mRNA exon-exon junctions. EMBO J 19: 6860–6869. 10.1093/emboj/19.24.6860 11118221 PMC305905

[bib48] Le Hir H, Saulière J, Wang Z (2016) The exon junction complex as a node of post-transcriptional networks. Nat Rev Mol Cell Biol 17: 41–54. 10.1038/nrm.2015.7 26670016

[bib49] Lee HC, Oh N, Cho H, Choe J, Kim YK (2010) Nonsense-mediated translational repression involves exon junction complex downstream of premature translation termination codon. FEBS Lett 584: 795–800. 10.1016/j.febslet.2010.01.003 20067791

[bib50] Lindeboom RG, Supek F, Lehner B (2016) The rules and impact of nonsense-mediated mRNA decay in human cancers. Nat Genet 48: 1112–1118. 10.1038/ng.3664 27618451 PMC5045715

[bib51] Losson R, Lacroute F (1979) Interference of nonsense mutations with eukaryotic messenger RNA stability. Proc Natl Acad Sci U S A 76: 5134–5137. 10.1073/pnas.76.10.5134 388431 PMC413094

[bib52] Lugowski A, Nicholson B, Rissland OS (2018) Determining mRNA half-lives on a transcriptome-wide scale. Methods 137: 90–98. 10.1016/j.ymeth.2017.12.006 29247756

[bib53] Lykke-Andersen J, Shu MD, Steitz JA (2001) Communication of the position of exon-exon junctions to the mRNA surveillance machinery by the protein RNPS1. Science 293: 1836–1839. 10.1126/science.1062786 11546874

[bib54] Lykke-Andersen S, Jensen TH (2015) Nonsense-mediated mRNA decay: An intricate machinery that shapes transcriptomes. Nat Rev Mol Cell Biol 16: 665–677. 10.1038/nrm4063 26397022

[bib55] Maquat LE, Kinniburgh AJ, Rachmilewitz EA, Ross J (1981) Unstable beta-globin mRNA in mRNA-deficient beta o thalassemia. Cell 27: 543–553. 10.1016/0092-8674(81)90396-2 6101206

[bib56] Mendell JT, Sharifi NA, Meyers JL, Martinez-Murillo F, Dietz HC (2004) Nonsense surveillance regulates expression of diverse classes of mammalian transcripts and mutes genomic noise. Nat Genet 36: 1073–1078. 10.1038/ng1429 15448691

[bib57] Miller JN, Pearce DA (2014) Nonsense-mediated decay in genetic disease: Friend or foe? Mutat Res Rev Mutat Res 762: 52–64. 10.1016/j.mrrev.2014.05.001 25485595 PMC4260155

[bib58] Muhlrad D, Parker R (1999) Recognition of yeast mRNAs as “nonsense containing” leads to both inhibition of mRNA translation and mRNA degradation: Implications for the control of mRNA decapping. Mol Biol Cell 10: 3971–3978. 10.1091/mbc.10.11.3971 10564284 PMC25692

[bib59] Nagy E, Maquat LE (1998) A rule for termination-codon position within intron-containing genes: When nonsense affects RNA abundance. Trends Biochem Sci 23: 198–199. 10.1016/s0968-0004(98)01208-0 9644970

[bib60] Natsume T, Kiyomitsu T, Saga Y, Kanemaki MT (2016) Rapid protein depletion in human cells by auxin-inducible degron tagging with short homology donors. Cell Rep 15: 210–218. 10.1016/j.celrep.2016.03.001 27052166

[bib61] Nickless A, Jackson E, Marasa J, Nugent P, Mercer RW, Piwnica-Worms D, You Z (2014) Intracellular calcium regulates nonsense-mediated mRNA decay. Nat Med 20: 961–966. 10.1038/nm.3620 25064126 PMC4126864

[bib62] Paillusson A, Hirschi N, Vallan C, Azzalin CM, Mühlemann O (2005) A GFP-based reporter system to monitor nonsense-mediated mRNA decay. Nucleic Acids Res 33: e54. 10.1093/nar/gni052 15800205 PMC1072805

[bib63] Palacios IM, Gatfield D, St Johnston D, Izaurralde E (2004) An eIF4AIII-containing complex required for mRNA localization and nonsense-mediated mRNA decay. Nature 427: 753–757. 10.1038/nature02351 14973490

[bib64] Pereverzev AP, Gurskaya NG, Ermakova GV, Kudryavtseva EI, Markina NM, Kotlobay AA, Lukyanov SA, Zaraisky AG, Lukyanov KA (2015) Method for quantitative analysis of nonsense-mediated mRNA decay at the single cell level. Sci Rep 5: 7729. 10.1038/srep07729 25578556 PMC4289894

[bib65] Rufener SC, Mühlemann O (2013) eIF4E-bound mRNPs are substrates for nonsense-mediated mRNA decay in mammalian cells. Nat Struct Mol Biol 20: 710–717. 10.1038/nsmb.2576 23665581

[bib66] Sato H, Singer RH (2021) Cellular variability of nonsense-mediated mRNA decay. BioRxiv. 10.1101/2021.03.31.437867. (Preprint posted March 31, 2021). PMC8664836 34893608

[bib67] Schlautmann LP, Gehring NH (2020) A day in the life of the exon junction complex. Biomolecules 10: 866. 10.3390/biom10060866 PMC7355637 32517083

[bib68] Sherf BA, Navarro SL, Hannah RR, Wood KV (1996) Dual-luciferase reporter assay: An advanced co-reporter technology integrating firefly and renilla luciferase assays. Promega Notes 57: 2–8.

[bib69] Sheth U, Parker R (2006) Targeting of aberrant mRNAs to cytoplasmic processing bodies. Cell 125: 1095–1109. 10.1016/j.cell.2006.04.037 16777600 PMC1858659

[bib70] Shibuya T, Tange TØ, Sonenberg N, Moore MJ (2004) eIF4AIII binds spliced mRNA in the exon junction complex and is essential for nonsense-mediated decay. Nat Struct Mol Biol 11: 346–351. 10.1038/nsmb750 15034551

[bib71] Tani H, Imamachi N, Salam KA, Mizutani R, Ijiri K, Irie T, Yada T, Suzuki Y, Akimitsu N (2012) Identification of hundreds of novel UPF1 target transcripts by direct determination of whole transcriptome stability. RNA Biol 9: 1370–1379. 10.4161/rna.22360 23064114 PMC3597577

[bib72] Trcek T, Sato H, Singer RH, Maquat LE (2013) Temporal and spatial characterization of nonsense-mediated mRNA decay. Genes Dev 27: 541–551. 10.1101/gad.209635.112 23431032 PMC3605467

[bib73] Ye J, She X, Liu Z, He Z, Gao X, Lu L, Liang R, Lin Y (2021) Eukaryotic initiation factor 4A-3: A review of its physiological role and involvement in oncogenesis. Front Oncol 11: 712045. 10.3389/fonc.2021.712045 34458150 PMC8386015

[bib74] You KT, Li LS, Kim NG, Kang HJ, Koh KH, Chwae YJ, Kim KM, Kim YK, Park SM, Jang SK, (2007) Selective translational repression of truncated proteins from frameshift mutation-derived mRNAs in tumors. PLoS Biol 5: e109. 10.1371/journal.pbio.0050109 17456004 PMC1854916

[bib75] Zhang J, Sun X, Qian Y, Maquat LE (1998) Intron function in the nonsense-mediated decay of beta-globin mRNA: Indications that pre-mRNA splicing in the nucleus can influence mRNA translation in the cytoplasm. RNA 4: 801–815. 10.1017/s1355838298971849 9671053 PMC1369660

[bib76] Zinshteyn B, Sinha NK, Enam SU, Koleske B, Green R (2021) Translational repression of NMD targets by GIGYF2 and EIF4E2. PLoS Genet 17: e1009813. 10.1371/journal.pgen.1009813 34665823 PMC8555832

