# Supplementary figures and images for: Nonsense-mediated mRNA decay uses complementary mechanisms to suppress mRNA and protein accumulation
Source: Life Sci Alliance. 2021 Dec 8;5(3):e202101217. doi: 10.26508/lsa.202101217 (PMC8711849; doi:10.26508/lsa.202101217)

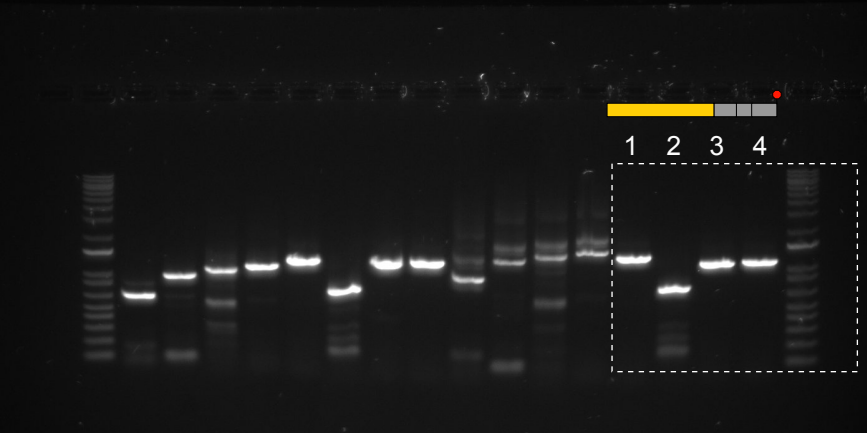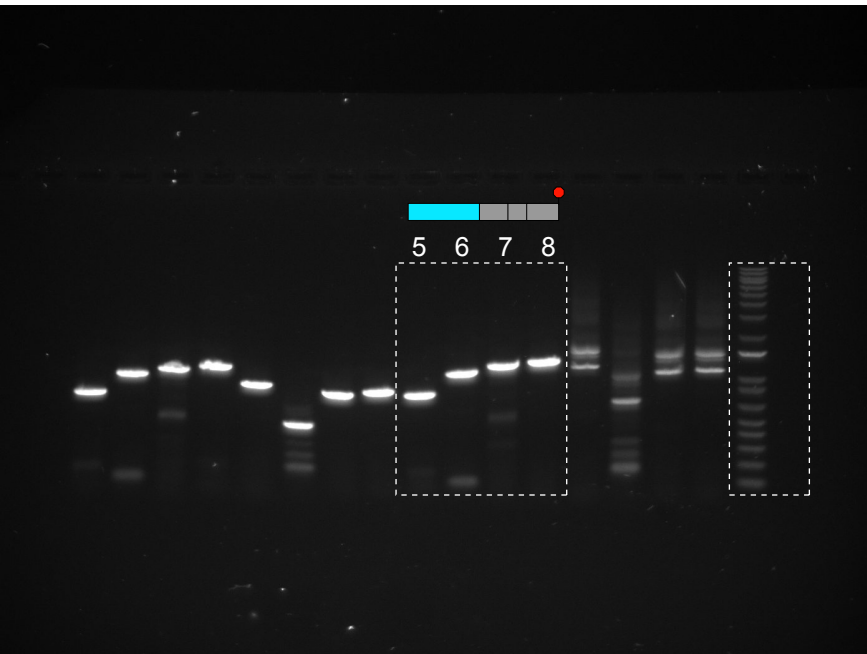

Supplement: Supplementary file 1 [file LSA-2021-01217_SdataFS1.pdf]

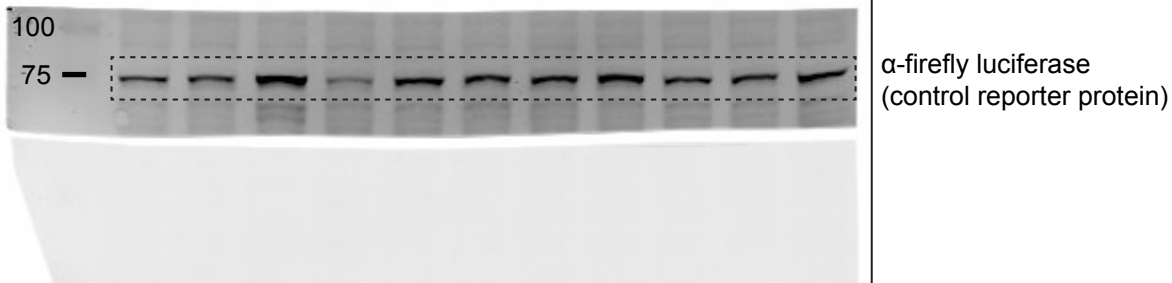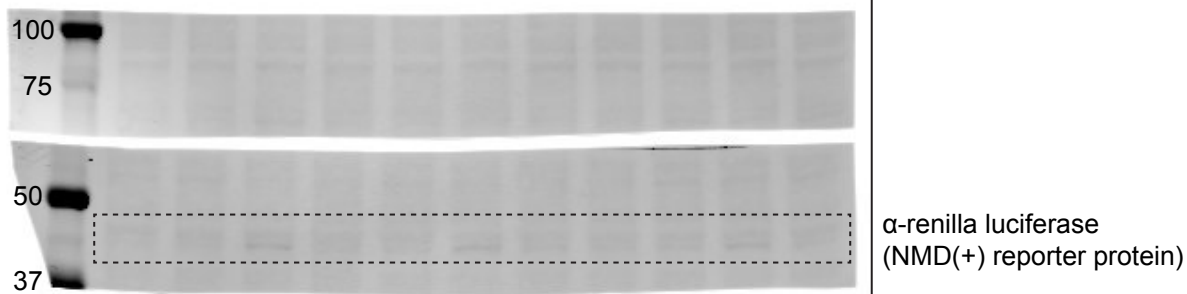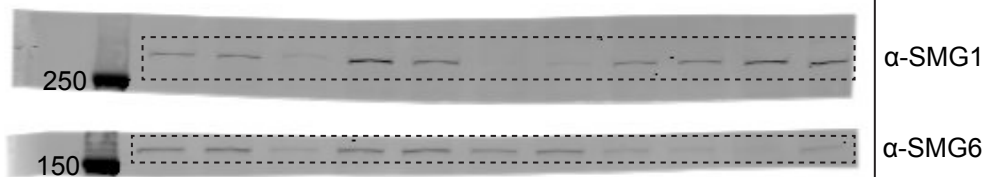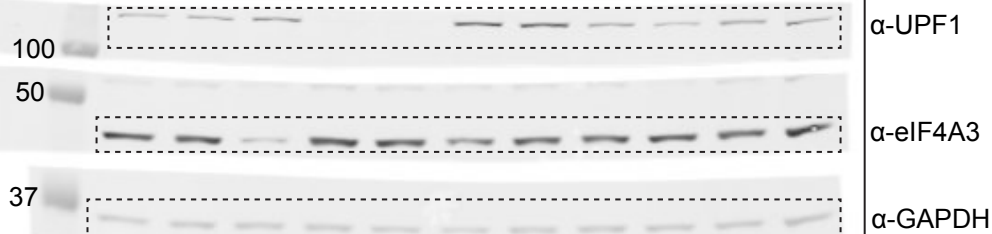

Supplement: Supplementary file 2 [file LSA-2021-01217_SdataFS2.pdf]

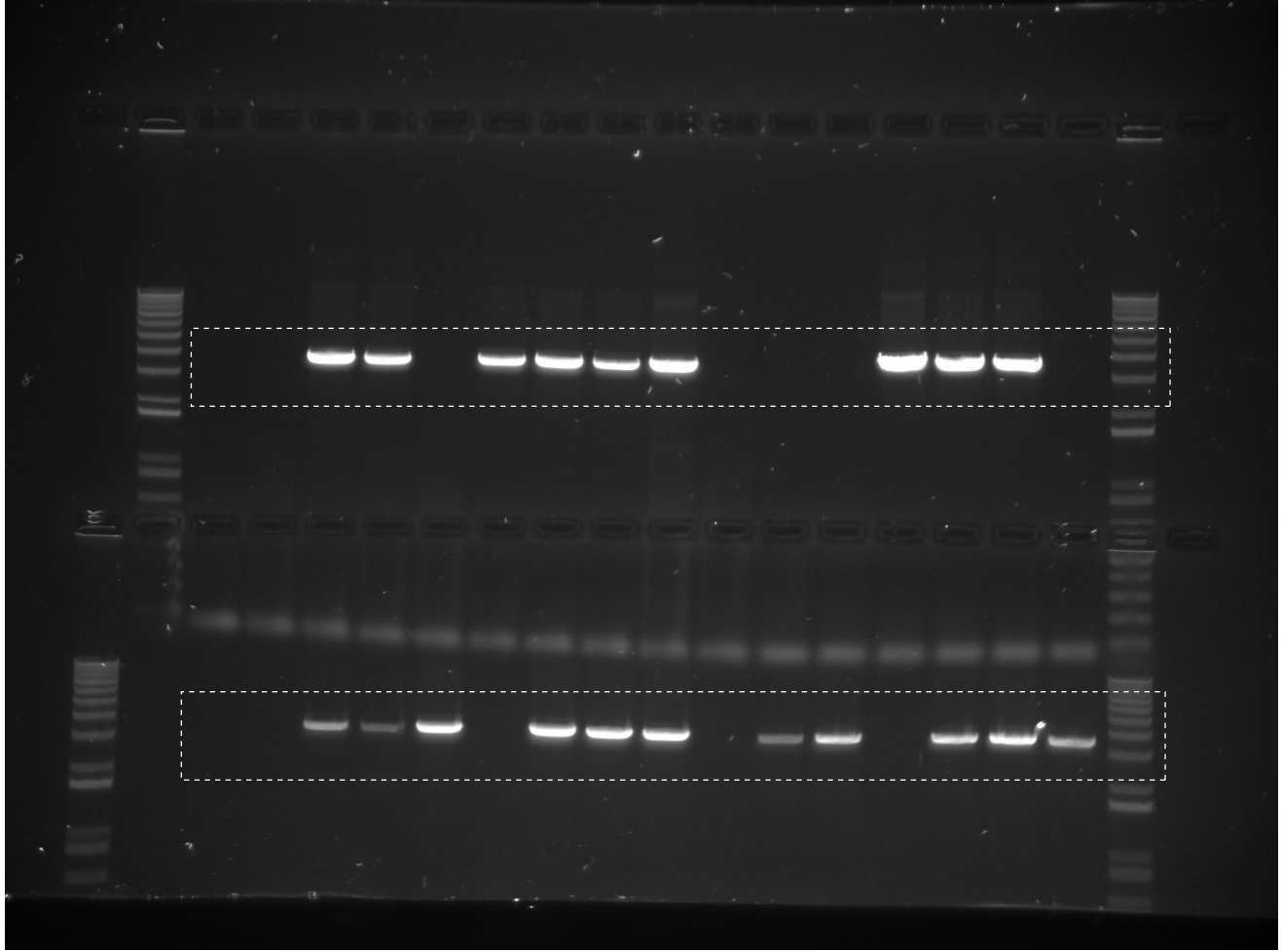

Supplement: Supplementary file 3 [file LSA-2021-01217_SdataFS3.pdf]

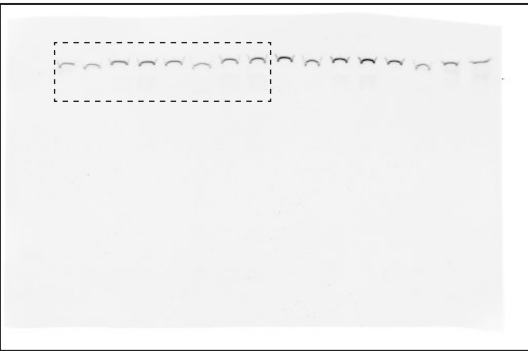

α-firefly luciferase

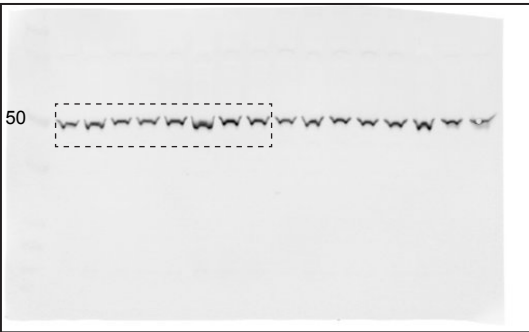

α-tubulin

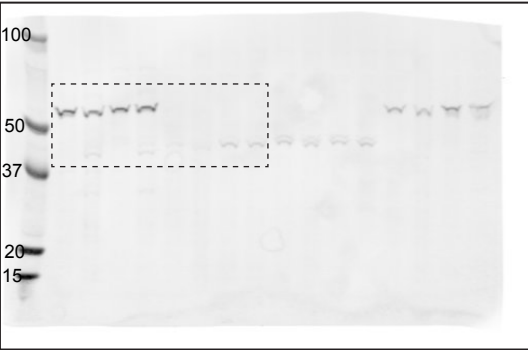

α-renilla luciferase

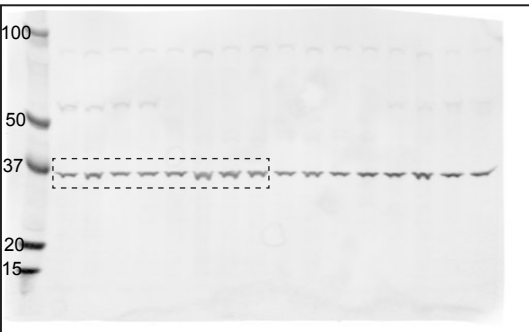

α-GAPDH

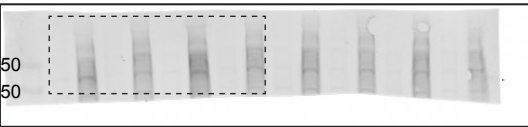

α-ubiquitin

Supplement: Supplementary file 4 [file LSA-2021-01217_SdataFS4.pdf]
