# Supplementary material for: Nonsense-mediated mRNA decay uses complementary mechanisms to suppress mRNA and protein accumulation
Source: Life Sci Alliance. 2021 Dec 8;5(3):e202101217. doi: 10.26508/lsa.202101217 (PMC8711849; doi:10.26508/lsa.202101217)
Supplement: Supplementary file 5 [file LSA-2021-01217_TableS1.docx]

Table 1. Key Resources Table.

| <!--Col Count:5-->Reagent type (species) or resource | Designation | Source or reference | Identifiers | Additional information |
| --- | --- | --- | --- | --- |
| Cell line (*Homo sapiens*) | Flp-In T-REx 293 Cell Line | Invitrogen | Cat. No. R78007 | Used for stable integration of all NMD reporters |
| Cell line (*H. sapiens*) | Control cell line; ctrl_1 | This paper |  | Control firefly and control renilla reporters stably integrated at AAVS1 loci; same reporters as ctrl_2 but created separately (biological replicate); polyclonal |
| Cell line (*H. sapiens*) | Control cell line; ctrl_2 | This paper |  | Control firefly and control renilla reporters stably integrated at AAVS1 loci; same reporters as ctrl_1 but created separately (biological replicate); polyclonal |
| Cell line (*H. sapiens*) | Renilla NMD(+) cell line; nmd_1 | This paper |  | Control firefly and NMD(+) renilla reporters stably integrated at AAVS1 loci; polyclonal |
| Cell line (*H. sapiens*) | Firefly NMD(+) cell line; nmd_2 | This paper |  | Control renilla and NMD(+) firefly reporters stably integrated at AAVS1 loci; polyclonal |
| Cell line (*H. sapiens*) | Monoclonal control cell line; ctrl_1-clone_1.2 | This paper |  | Control firefly and control renilla reporters stably integrated at AAVS1 loci; polyclonal cell line ctrl_1 underwent single cell sorting to get this cell line; monoclonal |
| Cell line (*H. sapiens*) | Monoclonal renilla NMD(+) cell line; nmd_1-clone_2.3 | This paper |  | Control firefly and NMD(+) renilla reporters stably integrated at AAVS1 loci; polyclonal cell line nmd_1 underwent single cell sorting to get this cell line; monoclonal |
| Cell line (*H. sapiens*) | Monoclonal renilla NMD(+) cell line; nmd_1-clone_2.4 | This paper |  | Control firefly and NMD(+) renilla reporters stably integrated at AAVS1 loci; polyclonal cell line nmd_1 underwent single cell sorting to get this cell line; monoclonal |
| Cell line (*H. sapiens*) | Monoclonal firefly NMD(+) cell line; nmd_2-clone_6.10 | This paper |  | Control renilla and NMD(+) firefly reporters stably integrated at AAVS1 loci; polyclonal cell line nmd_2 underwent single cell sorting to get this cell line; monoclonal |
| Recombinant DNA reagent | pCMV-3XFLAG- firefly-luciferase-beta-globin-control | PMID: 29528287 | Addgene ID 112085; RRID:Addgene_112085 | Transient transfection plasmid used to make renilla versions of the reporters and cloned into donor plasmid backbone for stable integration |
| Recombinant DNA reagent | pCMV-3XFLAG-firefly-luciferase-beta-globin-(39PTC) | PMID: 29528287 | Addgene ID 112084; RRID:Addgene_112084 | Transient transfection plasmid used to make renilla versions of the reporters and cloned into donor plasmid backbone for stable integration |
| Recombinant DNA reagent | pCMV-3XFLAG- renilla-luciferase-beta-globin-control | This paper | pRKB452 | Transient transfection plasmid cloned into donor plasmid backbone for stable integration; renilla sequence replaces firefly sequence in plasmid 112085 |
| Recombinant DNA reagent | pCMV-3XFLAG-renilla-luciferase-beta-globin-(39PTC) | This paper | pRKB453 | Transient transfection plasmid cloned into donor plasmid backbone for stable integration; renilla sequence replaces firefly sequence in plasmid 112084 |
| Recombinant DNA reagent | AAVS1-Tet-OsTIR1-PURO-AAVS1 | PMID: 27052166 | Addgene ID 72835; RRID:Addgene_72835 | Donor plasmid backbone for stable integration of NMD reporters; TIR1 sequence replaced with luciferase-beta-globin sequences from transient transfection constructs |
| Recombinant DNA reagent | AAVS1-TetOn-3XFLAG-firefly-beta-globin-control-AAVS1 | This paper | pRKB458 | Donor plasmid for stable integration of firefly control reporter; in cell lines ctrl_1, ctrl_2, nmd_1 |
| Recombinant DNA reagent | AAVS1-TetOn-3XFLAG-renilla-beta-globin-control-AAVS1 | This paper | pRKB460 | Donor plasmid for stable integration of renilla control reporter; in cell lines ctrl_1, ctrl_2, nmd_2 |
| Recombinant DNA reagent | AAVS1-TetOn-3XFLAG-firefly-beta-globin-PTC39-AAVS1 | This paper | pRKB459 | Donor plasmid for stable integration of firefly PTC39 reporter; in cell line nmd_2 |
| Recombinant DNA reagent | AAVS1-TetOn-3XFLAG-renilla-beta-globin-PTC39-AAVS1 | This paper | pRKB461 | Donor plasmid for stable integration of renilla PTC39 reporter; in cell line nmd_1 |
| Recombinant DNA reagent | pX459-sgAAVS1; Cas9/AAVS1-sgRNA | This paper; PMID: 24157548 | pRKB331 | pX459 (sgRNA and Cas9 expressing plasmid, RRID:Addgene_62988) with targeting sequence specific to AAVS1; targeting sequence is: GGGGCCACTAGGGACAGGAT |
| siRNA | siCtrl; non-targeting control siRNA | Dharmacon | D-001810-01-05 | ON-TARGETplus Non-targeting siRNA #1 |
| siRNA | siUpf1 #5 | Dharmacon | J-011763-05-0002 | ON-TARGETplus siRNA |
| siRNA | siUpf1 #7 | Dharmacon | J-011763-07-0002 | ON-TARGETplus siRNA |
| siRNA | siSmg1 #5 | Dharmacon | J-005033-05-0002 | ON-TARGETplus siRNA |
| siRNA | siSmg1 #7 | Dharmacon | J-005033-07-0002 | ON-TARGETplus siRNA |
| siRNA | siSmg6 #9 | Dharmacon | J-017845-09-0002 | ON-TARGETplus siRNA |
| siRNA | siSmg6 #10 | Dharmacon | J-017845-10-0002 | ON-TARGETplus siRNA |
| siRNA | siSmg6 #11 | Dharmacon | J-017845-11-0002 | ON-TARGETplus siRNA |
| siRNA | siSmg6 #12 | Dharmacon | J-017845-12-0002 | ON-TARGETplus siRNA |
| Sequenced-based reagent | sieIF4A3 | Thermo Fisher Scientific | Assay ID s18877; Cat. No. 4392420 | Silencer Select siRNA |
| Sequenced-based reagent | RKB3257 | This paper | PCR primer | AGCTTGCGGCCGCGAATTCAACTTCGAAAGTTTATGATCCAGAAC |
| Sequenced-based reagent | RKB3258 | This paper | PCR primer | AGATCTATCGATGAATTCGCTTGTTCATTTTTGAGAACTCGCTC |
| Sequenced-based reagent | RKB3259 | This paper | PCR primer | AGGATGACGATGACAAGCTTGCGGCCGCGAATTCAACTTCGAAAGTTTATGATCCAGAAC |
| Sequenced-based reagent | RKB3260 | This paper | PCR primer | CAAACTTGTTGATATCAGATCTATCGATGAATTCGCTTGTTCATTTTTGAGAACTCGCTC |
| Sequenced-based reagent | RKB3454 | This paper | PCR primer | TACCACTTCCTACCCTCGTAAAGAATTCGCGGCCGCAACCGTCAGAATTAACCATGGACT |
| Sequenced-based reagent | RKB3455 | This paper | PCR primer | GTGGTATGGCTGATTATGATCCTCTAGACATATGCTGCAGAACAAGAAAGCTGGGTCGGC |
| Sequenced-based reagent | RKB2250 | This paper | PCR primer | CGACTGAAATCCCTGGTAATC |
| Sequenced-based reagent | RKB2251 | This paper | PCR primer | CTACCGTGGTGTTCGTTTC |
| Sequenced-based reagent | RKB3600 | This paper | RT-PCR primer | GGCCTCGTGAAATCCCGTTA |
| Sequenced-based reagent | RKB3601 | This paper | RT-PCR primer | GAACAAGAAAGCTGGGTCGG |
| Sequenced-based reagent | RKB3602 | This paper | RT-PCR primer | GATTGGGGTGCTTGTTTGGC |
| Sequenced-based reagent | RKB3603 | This paper | RT-PCR primer | GCTGCAGAACAAGAAAGCTGG |
| Sequenced-based reagent | RKB3604 | This paper | RT-PCR primer | GGGCAAATCAGGCAAATCTGG |
| Sequenced-based reagent | RKB3605 | This paper | RT-PCR primer | AAGAAAGCTGGGTCGGCG |
| Sequenced-based reagent | RKB3606 | This paper | RT-PCR primer | GCCAGTAGCGCGGTGTATTA |
| Sequenced-based reagent | RKB3607 | This paper | RT-PCR primer | GCAGAACAAGAAAGCTGGGTC |
| Sequenced-based reagent | RKB3608 | This paper | RT-PCR primer | ATTACACCCGAGGGGGATGA |
| Sequenced-based reagent | RKB3609 | This paper | RT-PCR primer | GAAAAAGTTGCGCGGAGGAG |
| Sequenced-based reagent | RKB3610 | This paper | RT-PCR primer | GAGGCGAACTGTGTGTGAGA |
| Sequenced-based reagent | RKB3611 | This paper | RT-PCR primer | CTGGATACCGGGAAAACGCT |
| Sequenced-based reagent | RKB3612 | This paper | RT-PCR primer | ATGCTGCAGAACAAGAAAGCTG |
| Sequenced-based reagent | Firefly_1_F | PMID: 15451462 | qRT-PCR primer | AACATAAAGAAAGGCCCGGC |
| Sequenced-based reagent | Firefly_1_R | PMID: 15451462 | qRT-PCR primer | GCCTTATGCAGTTGCTCTCCA |
| Sequenced-based reagent | Firefly_2_F | PMID: 26252791 | qRT-PCR primer | GAAAGGCCCGGCGCCATTCT |
| Sequenced-based reagent | Firefly_2_R | PMID: 26252791 | qRT-PCR primer | TTCATAGCTTCTGCCAACCG |
| Sequenced-based reagent | Renilla_1_F | PMID: 20582318 | qRT-PCR primer | ACATGGTAACGCGGCCTCTT |
| Sequenced-based reagent | Renilla_1_R | PMID: 20582318 | qRT-PCR primer | TGCCCATACCAATAAGGTCTGGTA |
| Sequenced-based reagent | Renilla_2_F | This paper | qRT-PCR primer | GGGTGCTTGTTTGGCATTTC |
| Sequenced-based reagent | Renilla_2_R | This paper | qRT-PCR primer | AGGCCATTCATCCCATGATTC |
| Sequenced-based reagent | Rpl27_F | PMID: 28669802 | qRT-PCR primer | GCAAGAAGAAGATCGCCAAG |
| Sequenced-based reagent | Rpl27_R | PMID: 28669802 | qRT-PCR primer | TCCAAGGGGATATCCACAGA |
| Sequenced-based reagent | Srp14_F | PMID: 28669802 | qRT-PCR primer | GAGAGCGAGCAGTTCCTGAC |
| Sequenced-based reagent | Srp14_R | PMID: 28669802 | qRT-PCR primer | GTTTGGTTCGACCGTCATACT |
| Sequenced-based reagent | RKB2392 | This paper | Genomic DNA PCR primer | TTCCGCATTGGAGTCGCTTT |
| Sequenced-based reagent | RKB3517 | This paper | Genomic DNA PCR primer | GGTTCCATCTTCCAGCGGAT |
| Sequenced-based reagent | RKB3531 | This paper | Genomic DNA PCR primer | CATCCGTTTCCTTTGTTCTGGA |
| Antibody | Anti-firefly-luciferase (mouse monoclonal) | Abcam | ab16466; RRID:AB_443388 | 1:2,000 dilution clone Luci17 |
| Antibody | Anti-renilla-luciferase (rabbit monoclonal) | Abcam | Ab185926 | 1:2,000 dilution clone EPR17792 |
| Antibody | Anti-UPF1 (rabbit monoclonal) | Abcam | ab109363; RRID:AB_10861979 | 1:5,000 dilution clone EPR4681 |
| Antibody | Anti-eIF4A3 (rabbit monoclonal) | Abcam | ab180573 | 1:2,000 dilution clone EPR14301(B) |
| Antibody | Anti-SMG1 (rabbit polyclonal) | Bethyl Laboratories | A301-535A | 1:1,000 dilution |
| Antibody | Anti-Smg6 (rabbit polyclonal) | Abcam | ab87539 | 1:2,000 dilution |
| Antibody | Anti-GAPDH (rabbit polyclonal) | Bethyl Laboratories | A300-639A | 1:4,000 dilution |
| Antibody | Anti-ubiquitin (mouse monoclonal) | BostonBiochem | A-104 | 1:5,000 dilution clone 83406 |
| Antibody | Anti-alpha-tubulin | Sigma-Aldrich | T8203 | 1:5,000 clone AA13 |
| Antibody | IRDye 680RD Goat anti-Rabbit IgG Secondary Antibody | LI-COR | P/N: 926-68071; RRID:AB_10956166 | 1:10,000 dilution |
| Antibody | IRDye 800CW Donkey anti-Mouse IgG Secondary Antibody | LI-COR | P/N: 926-32212; RRID:AB_621847 | 1:10,000 dilution |
