# Supplementary material for: Nonsense-mediated mRNA decay uses complementary mechanisms to suppress mRNA and protein accumulation
Source: Life Sci Alliance. 2021 Dec 8;5(3):e202101217. doi: 10.26508/lsa.202101217 (PMC8711849; doi:10.26508/lsa.202101217)
Supplement: Supplementary file 6 [file LSA-2021-01217_TableS2.docx]

Table 2. Statistical significance values.

| <!--Col Count:5-->Figure | Cell line | siRNA | Comparison | *P*-value |
| --- | --- | --- | --- | --- |
| 2A | Firefly NMD(+) | Control | Firefly NMD(+) reporter versus firefly control reporter | 6.6 × 10^−12^ |
| 2A | Renilla NMD(+) | Control | Renilla NMD(+) reporter versus renilla control reporter | 1.1 × 10^−12^ |
| 2A | Firefly NMD(+) |  | Control versus eIF4A3 siRNA | 2.2 × 10^−9^ |
| 2A | Renilla NMD(+) |  | Control versus eIF4A3 siRNA | 3.8 × 10^−9^ |
| 2B | Firefly NMD(+) |  | Firefly NMD(+) versus control, 2 h | 8.5 × 10^−4^ |
| 2B | Firefly NMD(+) |  | Firefly NMD(+) versus control, 4 h | 7.6 × 10^−5^ |
| 2B | Renilla NMD(+) |  | Renilla NMD(+) versus control, 2 h | 1.8 × 10^−7^ |
| 2B | Renilla NMD(+) |  | Renilla NMD(+) versus control, 4 h | 2.6 × 10^−7^ |
| 3A | Firefly NMD(+) | Upf1 #5 | Firefly NMD(+) reporter versus firefly control reporter | 9.92 × 10^−6^ |
| 3A | Firefly NMD(+) | Upf1 #7 | Firefly NMD(+) reporter versus firefly control reporter | .0138 |
| 3A | Renilla NMD(+) | Upf1 #5 | Renilla NMD(+) reporter versus renilla control reporter | 2.21 × 10^−7^ |
| 3A | Renilla NMD(+) | Upf1 #7 | Renilla NMD(+) reporter versus renilla control reporter | 5.33 × 10^−7^ |
| 3A | Firefly NMD(+) | Smg1 #5 | Firefly NMD(+) reporter versus firefly control reporter | 1.28 × 10^−7^ |
| 3A | Firefly NMD(+) | Smg1 #7 | Firefly NMD(+) reporter versus firefly control reporter | 2.63 × 10^−5^ |
| 3A | Renilla NMD(+) | Smg1 #5 | Renilla NMD(+) reporter versus renilla control reporter | 1.37 × 10^−9^ |
| 3A | Renilla NMD(+) | Smg1 #7 | Renilla NMD(+) reporter versus renilla control reporter | 3.31 × 10^−5^ |
| 3A | Firefly NMD(+) | Smg6 #9 | Firefly NMD(+) reporter versus firefly control reporter | 0.09 |
| 3A | Firefly NMD(+) | Smg6 #10 | Firefly NMD(+) reporter versus firefly control reporter | 2.30 × 10^−5^ |
| 3A | Firefly NMD(+) | Smg6 #11 | Firefly NMD(+) reporter versus firefly control reporter | 2.50 × 10^−3^ |
| 3A | Firefly NMD(+) | Smg6 #12 | Firefly NMD(+) reporter versus firefly control reporter | 0.108 |
| 3A | Renilla NMD(+) | Smg6 #9 | Renilla NMD(+) reporter versus renilla control reporter | 0.018 |
| 3A | Renilla NMD(+) | Smg6 #10 | Renilla NMD(+) reporter versus renilla control reporter | 6.62 × 10^−5^ |
| 3A | Renilla NMD(+) | Smg6 #11 | Renilla NMD(+) reporter versus renilla control reporter | 4.43 × 10^−4^ |
| 3A | Renilla NMD(+) | Smg6 #12 | Renilla NMD(+) reporter versus renilla control reporter | 4.39 × 10^−8^ |
| 3A | Firefly NMD(+) | eIF4A3 | Firefly NMD(+) reporter versus firefly control reporter | 5.27 × 10^−9^ |
| 3A | Renilla NMD(+) | eIF4A3 | Renilla NMD(+) reporter versus renilla control reporter | 2.29 × 10^−9^ |
| 3B | Firefly NMD(+) | control | RNA versus protein | 4.87 × 10^−8^ |
| 3B | Firefly NMD(+) | Smg1 #5 | RNA versus protein | 9.27 × 10^−10^ |
| 3B | Firefly NMD(+) | Smg6 #11 | RNA versus protein | 0.00133 |
| 3B | Firefly NMD(+) | eIF4A3 | RNA versus protein | 3.12 × 10^−6^ |
| 3B | Renilla NMD(+) | control | RNA versus protein | 8.51 × 10^−11^ |
| 3B | Renilla NMD(+) | Smg1 #5 | RNA versus protein | 2.88 × 10^−10^ |
| 3B | Renilla NMD(+) | Smg6 #11 | RNA versus protein | 0.00273 |
| 3B | Renilla NMD(+) | eIF4A3 | RNA versus protein | 3.72 × 10^−14^ |
| 4B | Firefly NMD(+) |  | Firefly NMD(+) versus control, 30 min | 0.76 |
| 4B | Firefly NMD(+) |  | Firefly NMD(+) versus control, 60 min | 0.76 |
| 4B | Firefly NMD(+) |  | Firefly NMD(+) versus control, 120 min | 0.12 |
| 4B | Renilla NMD(+) |  | Renilla NMD(+) versus control, 30 min | 0.18 |
| 4B | Renilla NMD(+) |  | Renilla NMD(+) versus control, 60 min | 0.0099 |
| 4B | Renilla NMD(+) |  | Renilla NMD(+) versus control, 120 min | 0.0014 |
| 4C | Firefly NMD(+) | Control | +/−MG132 | 0.62 |
| 4C | Firefly NMD(+) | eIF4A3 | +/−MG132 | 0.43 |
| 4C | Renilla NMD(+) | Control | +/−MG132 | 2.9 × 10^−4^ |
| 4C | Renilla NMD(+) | eIF4A3 | +/−MG132 | 0.71 |
| S3C | Firefly NMD(+) clone 6.10 |  | Firefly NMD(+) reporter versus firefly control reporter | 0.015 |
| S3C | Renilla NMD(+) clone 2.3 |  | Renilla NMD(+) reporter versus renilla control reporter | 2.7 × 10^−9^ |
| S3C | Renilla NMD(+) clone 2.4 |  | Renilla NMD(+) reporter versus renilla control reporter | 3.2 × 10^−9^ |
| S3E | Firefly NMD(+) | Upf1 #5 | RNA versus protein | 7.53 × 10^−13^ |
| S3E | Firefly NMD(+) | Upf1 #7 | RNA versus protein | 1.50 × 10^−12^ |
| S3E | Firefly NMD(+) | Smg1 #7 | RNA versus protein | 5.14 × 10^−6^ |
| S3E | Firefly NMD(+) | Smg6 #10 | RNA versus protein | 8.28 × 10^−11^ |
| S3E | Renilla NMD(+) | Upf1 #5 | RNA versus protein | 2.75 × 10^−12^ |
| S3E | Renilla NMD(+) | Upf1 #7 | RNA versus protein | 2.08 × 10^−14^ |
| S3E | Renilla NMD(+) | Smg1 #7 | RNA versus protein | 1.46 × 10^−7^ |
| S3E | Renilla NMD(+) | Smg6 #10 | RNA versus protein | 2.70 × 10^−13^ |
| S3G | Firefly NMD(+) |  | 24 versus 48 h | 0.28 |
| S3G | Firefly NMD(+) |  | 24 versus 72 h | 0.744 |
| S3G | Firefly NMD(+) |  | 24 versus 96 h | 0.76 |
| S3G | Firefly NMD(+) |  | 24 versus 120 h | 0.384 |
| S3G | Renilla NMD(+) |  | 24 versus 48 h | 0.576 |
| S3G | Renilla NMD(+) |  | 24 versus 72 h | 0.008 |
| S3G | Renilla NMD(+) |  | 24 versus 96 h | 1 |
| S3G | Renilla NMD(+) |  | 24 versus 120 h | 0.024 |
| S3H | Firefly NMD(+) |  | RNA versus protein, 24 h | 3.80 × 10^−9^ |
| S3H | Firefly NMD(+) |  | RNA versus protein, 120 h | 1.36 × 10^−7^ |
| S3H | Renilla NMD(+) |  | RNA versus protein, 24 h | 6.32 × 10^−11^ |
| S3H | Renilla NMD(+) |  | RNA versus protein, 120 h | 6.12 × 10^−9^ |
