# Supplementary material for: Nonsense-mediated mRNA decay uses complementary mechanisms to suppress mRNA and protein accumulation
Source: Life Sci Alliance. 2021 Dec 8;5(3):e202101217. doi: 10.26508/lsa.202101217 (PMC8711849; doi:10.26508/lsa.202101217)
Supplement: Supplementary file 7 [file LSA-2021-01217_TableS3.docx]

Table 3. Median box plot values as percentages.

| <!--Col Count:6-->Figure | Cell line | siRNA | Reporter | Variable | % |
| --- | --- | --- | --- | --- | --- |
| 2A | Firefly NMD(+) | Control | Firefly NMD(+) |  | 27.2 |
| 2A | Firefly NMD(+) | eIF4A3 | Firefly NMD(+) |  | 67.9 |
| 2A | Renilla NMD(+) | Control | Renilla NMD(+) |  | 15.7 |
| 2A | Renilla NMD(+) | eIF4A3 | Renilla NMD(+) |  | 24.9 |
| 2B | Control |  | Firefly control | 2 h | 96.5 |
| 2B | Control |  | Renilla control | 2 h | 94.0 |
| 2B | Control |  | Firefly control | 4 h | 42.3 |
| 2B | Control |  | Renilla control | 4 h | 48.7 |
| 2B | Firefly NMD(+) |  | Firefly NMD(+) | 2 h | 76.3 |
| 2B | Firefly NMD(+) |  | Renilla control | 2 h | 92.8 |
| 2B | Firefly NMD(+) |  | Firefly NMD(+) | 4 h | 29.9 |
| 2B | Firefly NMD(+) |  | Renilla control | 4 h | 53.0 |
| 2B | Renilla NMD(+) |  | Firefly control | 2 h | 90.8 |
| 2B | Renilla NMD(+) |  | Renilla NMD(+) | 2 h | 54.1 |
| 2B | Renilla NMD(+) |  | Firefly control | 4 h | 31.4 |
| 2B | Renilla NMD(+) |  | Renilla NMD(+) | 4 h | 19.0 |
| 3A | Firefly NMD(+) | Upf1 #5 | Firefly NMD(+) |  | 151 |
| 3A | Firefly NMD(+) | Upf1 #7 | Firefly NMD(+) |  | 122 |
| 3A | Renilla NMD(+) | Upf1 #5 | Renilla NMD(+) |  | 203 |
| 3A | Renilla NMD(+) | Upf1 #7 | Renilla NMD(+) |  | 219 |
| 3A | Firefly NMD(+) | Smg1 #5 | Firefly NMD(+) |  | 460 |
| 3A | Firefly NMD(+) | Smg1 #7 | Firefly NMD(+) |  | 211 |
| 3A | Renilla NMD(+) | Smg1 #5 | Renilla NMD(+) |  | 660 |
| 3A | Renilla NMD(+) | Smg1 #7 | Renilla NMD(+) |  | 240 |
| 3A | Firefly NMD(+) | Smg6 #9 | Firefly NMD(+) |  | 89.7 |
| 3A | Firefly NMD(+) | Smg6 #10 | Firefly NMD(+) |  | 147 |
| 3A | Firefly NMD(+) | Smg6 #11 | Firefly NMD(+) |  | 262 |
| 3A | Firefly NMD(+) | Smg6 #12 | Firefly NMD(+) |  | 116 |
| 3A | Renilla NMD(+) | Smg6 #9 | Renilla NMD(+) |  | 130 |
| 3A | Renilla NMD(+) | Smg6 #10 | Renilla NMD(+) |  | 138 |
| 3A | Renilla NMD(+) | Smg6 #11 | Renilla NMD(+) |  | 375 |
| 3A | Renilla NMD(+) | Smg6 #12 | Renilla NMD(+) |  | 139 |
| 3A | Firefly NMD(+) | eIF4A3 | Firefly NMD(+) |  | 573 |
| 3A | Renilla NMD(+) | eIF4A3 | Renilla NMD(+) |  | 744 |
| 3B | Firefly NMD(+) | Control | Firefly NMD(+) | RNA | 16.2 |
| 3B | Firefly NMD(+) | Control | Firefly NMD(+) | Protein | 5.59 |
| 3B | Firefly NMD(+) | Smg1 #5 | Firefly NMD(+) | RNA | 39.2 |
| 3B | Firefly NMD(+) | Smg1 #5 | Firefly NMD(+) | Protein | 27.4 |
| 3B | Firefly NMD(+) | Smg6 #11 | Firefly NMD(+) | RNA | 34.8 |
| 3B | Firefly NMD(+) | Smg6 #11 | Firefly NMD(+) | Protein | 15.6 |
| 3B | Firefly NMD(+) | eIF4A3 | Firefly NMD(+) | RNA | 41.2 |
| 3B | Firefly NMD(+) | eIF4A3 | Firefly NMD(+) | Protein | 34.1 |
| 3B | Renilla NMD(+) | Control | Renilla NMD(+) | RNA | 8.68 |
| 3B | Renilla NMD(+) | Control | Renilla NMD(+) | Protein | 2.32 |
| 3B | Renilla NMD(+) | Smg1 #5 | Renilla NMD(+) | RNA | 23.2 |
| 3B | Renilla NMD(+) | Smg1 #5 | Renilla NMD(+) | Protein | 14.8 |
| 3B | Renilla NMD(+) | Smg6 #11 | Renilla NMD(+) | RNA | 17.3 |
| 3B | Renilla NMD(+) | Smg6 #11 | Renilla NMD(+) | Protein | 8.40 |
| 3B | Renilla NMD(+) | eIF4A3 | Renilla NMD(+) | RNA | 35.7 |
| 3B | Renilla NMD(+) | eIF4A3 | Renilla NMD(+) | Protein | 16.7 |
| 4A | Control |  | Firefly control | 30 min | 80.8 |
| 4A | Control |  | Renilla control | 30 min | 82.4 |
| 4A | Control |  | Firefly control | 60 min | 82.5 |
| 4A | Control |  | Renilla control | 60 min | 79.5 |
| 4A | Control |  | Firefly control | 120 min | 64.9 |
| 4A | Control |  | Renilla control | 120 min | 58.8 |
| 4A | Control |  | Firefly control | 360 min | 34.1 |
| 4A | Control |  | Renilla control | 360 min | 27.7 |
| 4A | Firefly NMD(+) |  | Firefly NMD(+) | 30 min | 83.6 |
| 4A | Firefly NMD(+) |  | Renilla control | 30 min | 83 |
| 4A | Firefly NMD(+) |  | Firefly NMD(+) | 60 min | 70.6 |
| 4A | Firefly NMD(+) |  | Renilla control | 60 min | 69.7 |
| 4A | Firefly NMD(+) |  | Firefly NMD(+) | 120 min | 58.7 |
| 4A | Firefly NMD(+) |  | Renilla control | 120 min | 59.1 |
| 4A | Firefly NMD(+) |  | Firefly NMD(+) | 360 min | 28.2 |
| 4A | Firefly NMD(+) |  | Renilla control | 360 min | 29.3 |
| 4A | Renilla NMD(+) |  | Firefly control | 30 min | 83.9 |
| 4A | Renilla NMD(+) |  | Renilla NMD(+) | 30 min | 80.8 |
| 4A | Renilla NMD(+) |  | Firefly control | 60 min | 78.2 |
| 4A | Renilla NMD(+) |  | Renilla NMD(+) | 60 min | 66.5 |
| 4A | Renilla NMD(+) |  | Firefly control | 120 min | 72.5 |
| 4A | Renilla NMD(+) |  | Renilla NMD(+) | 120 min | 57 |
| 4A | Renilla NMD(+) |  | Firefly control | 360 min | 38.9 |
| 4A | Renilla NMD(+) |  | Renilla NMD(+) | 360 min | 38.5 |
| 4C | Firefly NMD(+) | Control | Firefly NMD(+) | −MG132 | 8.19 |
| 4C | Firefly NMD(+) | Control | Firefly NMD(+) | +MG132 | 8.76 |
| 4C | Firefly NMD(+) | eIF4A3 | Firefly NMD(+) | −MG132 | 50.7 |
| 4C | Firefly NMD(+) | eIF4A3 | Firefly NMD(+) | +MG132 | 45.8 |
| 4C | Renilla NMD(+) | Control | Renilla NMD(+) | −MG132 | 1.94 |
| 4C | Renilla NMD(+) | Control | Renilla NMD(+) | +MG132 | 3.77 |
| 4C | Renilla NMD(+) | eIF4A3 | Renilla NMD(+) | -MG132 | 14.7 |
| 4C | Renilla NMD(+) | eIF4A3 | Renilla NMD(+) | +MG132 | 14.0 |
| S3C | Firefly NMD(+) clone 6.10 |  | Firefly NMD(+) |  | 15.4 |
| S3C | Renilla NMD(+) clone 2.3 |  | Renilla NMD(+) |  | 1.36 |
| S3C | Renilla NMD(+) clone 2.4 |  | Renilla NMD(+) |  | 1.10 |
| S3E | Firefly NMD(+) | Control | Firefly NMD(+) | RNA | 16.2 |
| S3E | Firefly NMD(+) | Control | Firefly NMD(+) | Protein | 5.59 |
| S3E | Firefly NMD(+) | Upf1 #5 | Firefly NMD(+) | RNA | 20.8 |
| S3E | Firefly NMD(+) | Upf1 #5 | Firefly NMD(+) | Protein | 8.99 |
| S3E | Firefly NMD(+) | Upf1 #7 | Firefly NMD(+) | RNA | 18.3 |
| S3E | Firefly NMD(+) | Upf1 #7 | Firefly NMD(+) | Protein | 7.23 |
| S3E | Firefly NMD(+) | Smg1 #7 | Firefly NMD(+) | RNA | 26.0 |
| S3E | Firefly NMD(+) | Smg1 #7 | Firefly NMD(+) | Protein | 12.5 |
| S3E | Firefly NMD(+) | Smg6 #10 | Firefly NMD(+) | RNA | 21.2 |
| S3E | Firefly NMD(+) | Smg6 #10 | Firefly NMD(+) | Protein | 8.75 |
| S3E | Renilla NMD(+) | Control | Renilla NMD(+) | RNA | 8.68 |
| S3E | Renilla NMD(+) | Control | Renilla NMD(+) | Protein | 2.32 |
| S3E | Renilla NMD(+) | Upf1 #5 | Renilla NMD(+) | RNA | 16.2 |
| S3E | Renilla NMD(+) | Upf1 #5 | Renilla NMD(+) | Protein | 4.57 |
| S3E | Renilla NMD(+) | Upf1 #7 | Renilla NMD(+) | RNA | 15.8 |
| S3E | Renilla NMD(+) | Upf1 #7 | Renilla NMD(+) | Protein | 4.95 |
| S3E | Renilla NMD(+) | Smg1 #7 | Renilla NMD(+) | RNA | 13.0 |
| S3E | Renilla NMD(+) | Smg1 #7 | Renilla NMD(+) | Protein | 5.41 |
| S3E | Renilla NMD(+) | Smg6 #10 | Renilla NMD(+) | RNA | 9.95 |
| S3E | Renilla NMD(+) | Smg6 #10 | Renilla NMD(+) | Protein | 3.11 |
| S3F | Control #1 |  | Firefly control | 48 h | 200 |
| S3F | Control #1 |  | Firefly control | 72 h | 167 |
| S3F | Control #1 |  | Firefly control | 96 h | 208 |
| S3F | Control #1 |  | Firefly control | 120 h | 236 |
| S3F | Control #2 |  | Firefly control | 48 h | 168 |
| S3F | Control #2 |  | Firefly control | 72 h | 159 |
| S3F | Control #2 |  | Firefly control | 96 h | 259 |
| S3F | Control #2 |  | Firefly control | 120 h | 264 |
| S3F | Firefly NMD(+) |  | Firefly NMD(+) | 48 h | 159 |
| S3F | Firefly NMD(+) |  | Firefly NMD(+) | 72 h | 215 |
| S3F | Firefly NMD(+) |  | Firefly NMD(+) | 96 h | 298 |
| S3F | Firefly NMD(+) |  | Firefly NMD(+) | 120 h | 338 |
| S3F | Renilla NMD(+) |  | Firefly control | 48 h | 199 |
| S3F | Renilla NMD(+) |  | Firefly control | 72 h | 170 |
| S3F | Renilla NMD(+) |  | Firefly control | 96 h | 210 |
| S3F | Renilla NMD(+) |  | Firefly control | 120 h | 240 |
| S3F | Control #1 |  | Renilla control | 48 h | 181 |
| S3F | Control #1 |  | Renilla control | 72 h | 185 |
| S3F | Control #1 |  | Renilla control | 96 h | 223 |
| S3F | Control #1 |  | Renilla control | 120 h | 264 |
| S3F | Control #2 |  | Renilla control | 48 h | 183 |
| S3F | Control #2 |  | Renilla control | 72 h | 196 |
| S3F | Control #2 |  | Renilla control | 96 h | 276 |
| S3F | Control #2 |  | Renilla control | 120 h | 294 |
| S3F | Firefly NMD(+) |  | Renilla control | 48 h | 199 |
| S3F | Firefly NMD(+) |  | Renilla control | 72 h | 246 |
| S3F | Firefly NMD(+) |  | Renilla control | 96 h | 305 |
| S3F | Firefly NMD(+) |  | Renilla control | 120 h | 336 |
| S3F | Renilla NMD(+) |  | Renilla NMD(+) | 48 h | 202 |
| S3F | Renilla NMD(+) |  | Renilla NMD(+) | 72 h | 162 |
| S3F | Renilla NMD(+) |  | Renilla NMD(+) | 96 h | 214 |
| S3F | Renilla NMD(+) |  | Renilla NMD(+) | 120 h | 208 |
| S3G | Firefly NMD(+) |  | Firefly NMD(+) | 24 h | 4.26 |
| S3G | Firefly NMD(+) |  | Firefly NMD(+) | 48 h | 3.6 |
| S3G | Firefly NMD(+) |  | Firefly NMD(+) | 72 h | 4.6 |
| S3G | Firefly NMD(+) |  | Firefly NMD(+) | 96 h | 4.78 |
| S3G | Firefly NMD(+) |  | Renilla NMD(+) | 120 h | 5.03 |
| S3G | Renilla NMD(+) |  | Renilla NMD(+) | 24 h | 3.53 |
| S3G | Renilla NMD(+) |  | Renilla NMD(+) | 48 h | 3.11 |
| S3G | Renilla NMD(+) |  | Renilla NMD(+) | 72 h | 2.77 |
| S3G | Renilla NMD(+) |  | Renilla NMD(+) | 96 h | 3.27 |
| S3G | Renilla NMD(+) |  | Renilla NMD(+) | 120 h | 2.68 |
| S3H | Firefly NMD(+) |  | Firefly NMD(+) | 24 h, RNA | 17.2 |
| S3H | Firefly NMD(+) |  | Firefly NMD(+) | 24 h, protein | 4.26 |
| S3H | Firefly NMD(+) |  | Firefly NMD(+) | 120 h, RNA | 17.6 |
| S3H | Firefly NMD(+) |  | Firefly NMD(+) | 120 h, protein | 5.03 |
| S3H | Renilla NMD(+) |  | Renilla NMD(+) | 24 h, RNA | 14.1 |
| S3H | Renilla NMD(+) |  | Renilla NMD(+) | 24 h, protein | 3.53 |
| S3H | Renilla NMD(+) |  | Renilla NMD(+) | 120 h, RNA | 10.7 |
| S3H | Renilla NMD(+) |  | Renilla NMD(+) | 120 h, protein | 2.68 |
